# Carbonized Polymer Dot‐Tannic Acid Nanoglue: Tissue Reinforcement with Concurrent Fluorescent Tracking, Insulin Delivery, and Reactive Oxygen Species Regulation for Normal and Diabetic Wound Healing

**DOI:** 10.1002/smll.202405531

**Published:** 2024-08-15

**Authors:** Maansi Aggarwal, Deepinder Sharda, Shruti Srivastava, Dinesh Kumar Kotnees, Diptiman Choudhury, Prolay Das

**Affiliations:** ^1^ Department of Chemistry Indian Institute of Technology Patna Patna Bihar 801103 India; ^2^ Department of Chemistry and Biochemistry Thapar Institute of Engineering and Technology (TIET) Patiala Punjab 147004 India; ^3^ Department of Metallurgical and Materials Engineering Indian Institute of Technology Patna Patna Bihar 801103 India; ^4^ Center of Excellence in Emerging Materials (CEEMS) Thapar Institute of Engineering and Technology Patiala Punjab 147004 India

**Keywords:** antibacterial photodynamic therapy, carbonized polymer dot, diabetic wound healing, nanoglue, ROS scavenger and generator

## Abstract

Nanotizing biosealant components offer a multitude of chemical functionalities for superior adhesion–cohesion, delivering unique properties for comprehensive wound healing that are otherwise impossible to achieve using commercial variants. For the first time, a two‐step controlled hydrothermal pyrolysis is reported to nanotize dopamine, phloroglucinol, and glutaraldehyde into carbon dot (CD) to be subsequently converted into carbonized polymer dot (CPD) with gelatin as a co‐substrate. Chemical crosslinking of CD with gelatin through Schiff base formation before the second pyrolysis step ensures a complex yet porous polymeric network. The retention of chemical functionalities indigenous to CD substrates and gelatin along with the preservation of CD photoluminescence in CPD for optical tracking is achieved. A unique nanoformulation is created with the CPD through tannic acid (TA) grafting evolving CPD‐TA nanoglue demonstrating ≈1.32 MPa strength in lap shear tests conducted on porcine skin, surpassing traditional bioadhesives. CPD‐TA nanoglue uploaded insulin as chosen cargo disbursal at the wound site for healing normal and in vitro diabetic wound models using HEKa cells with extraordinary biocompatibility. Most importantly, CPD‐TA can generate reactive oxygen species (ROS) and scavenge simultaneously under ambient conditions (23 W white LED or dark) for on‐demand sterilization or aiding wound recovery through ROS scavenging.

## Graphical abstract

The scheme displays a two‐step hydrothermal synthesis of carbonized polymer dot‐tannic acid (CPD‐TA) fluorescent nanoglue utilizing carbon dot as a crosslinking agent with gelatin to form CPD. The formed nanoglue can regulate reactive oxygen species under ambient conditions and demonstrates advanced adhesion strength of 1.32 MPa, surpassing traditional bioadhesives.

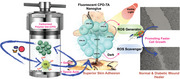

## 1 Introduction

Bioglue or biosealant are alternatives to sutures, staples, and patches designed to seal, adhere or reinforce tissues.^[^ ^1^ ^]^ Over the years few bioglue formulations have been approved for public use.^[^ ^2^ ^]^ Cyanoacrylates class of bioglue has been a popular market‐available product for quite some time.^[^ ^3^ ^]^ In some bioglues, proteins like Bovine Serum Albumin/Human Serum Albumin are crosslinked in situ with glutaraldehyde like polyaldehyde/polyethylene glycol or they may be protein‐free formulations of polyalcohols in assorted hydrogel and buffers.^[^ ^4^, ^5^, ^6^, ^7^ ^]^ However, the trend to get over aldehyde and isocyanate‐free bioglue has thrown fresh challenges to developing newer biosealant products with less toxicity associated with aldehydes and cyanoacrylates.^[^ ^8^ ^]^ The expedited post‐sealing wound healing process and infection prevention are other critical aspects of wound management, which becomes more challenging under certain disease conditions like diabetes.^[^ ^9^ ^]^ This demands comprehensive care from the bioglue itself, an aspect lacking in market‐available biosealants but intensely researched. Herein, we hypothesized the creation of a carbonized nanoparticle as a multifunctional crosslinking core to formulate a nontoxic comprehensive bioglue/nanoglue that decorates itself with the requisite functional groups vital for sealing and reinforcing tissues and is simultaneously capable of delivering drugs/growth factors with the provision of photoactivated self‐sterilization. The fact that the nanoglue can synergistically generate and scavenge reactive oxygen species (ROS) makes it an interesting and unique candidate among many of the recently reported entries of its kind.

Optimized adhesion–cohesion of bioglues with tissues including skin are corollary of swift reactions between chemical functional groups in the glue and tissues leading to effective crosslinking. Accountability of adhesion relies on the polyphenolic groups, amine, and aldehydes within the bioglue facilitating non‐covalent and covalent interactions like Michael addition, esterification, and Schiff base formation.^[^ ^10^, ^11^, ^12^ ^]^ Thus, presenting and offering the desired functional groups by the bioglue is key to optimal activity for which we sought chemical intervention in the nanoscale through the creation of carbon dots (CD). Manipulation of reaction conditions including time and temperature of pyrolysis can retain the functional groups of the substrates in CD, which plays pivotal roles in crosslinking, photoactivated energy/electron transfer, and interesting optoelectronic transitions. Such all‐in‐all properties are rare in other nanomaterials prompting researchers to demonstrate the wide applicability of CD in drug delivery, imaging, polymerization, energy harvesting, photocatalysis, sensing, and many others.^[^ ^13^ ^]^ Inspiringly, we hypothesized to employ CD derived from specific substrates to obtain favorable chemical outcomes for the desired adhesive application, one being the retention of reactive‐CHO functionality. The CD was further transformed into carbon polymer dots (CPD) where an acute balance between the carbonization and the polymerization stage is necessitated for CPD formation. Additive unsaturated bonds, crosslinkable active sites, and dehydratable functional group precursors aid their formation to offer greater tackiness in nanoglue compared to using a pristine CD.^[^ ^14^, ^15^ ^]^ Structurally, CPD interlinks the polymers and CD consisting of high crosslinking networks and trivial graphitization with a hydrophobic core in combination with formulated interweaving polymeric chains surface rendering more stability and biocompatibility than synthetic polymers.^[^ ^16^, ^17^, ^18^ ^]^ CPD broadcasts a broad substrate scope ranging from organic precursors to polymers prompting their potential application as light‐emitting diodes, imaging agents, anticorrosive agents, and lubricants, albeit not in the context of bioglue components.^[^ ^19^, ^20^, ^21^ ^]^

A two‐step controlled hydrothermal pyrolysis is demonstrated for the first time to generate CD and subsequently CPD applicable to this nanoglue formulation in co‐conjugation with gelatin, an inexpensive edible biomass derivable protein. Conjecturing the translation of the superior biocompatibility and biodegradability of gelatin,^[^ ^22^ ^]^ its involvement in the second pyrolysis step is a novel approach to optimize the adhesion–cohesion properties of the nanoglue through the retention of functional groups of both the precursor CD and gelatin in the CPDs. To establish a delicate balance between cohesion, adhesion, and trackable photoluminescence (PL) of the CPD, the post‐synthetic addition of tannic acid (TA) was found to be key in achieving the desired nanoglue formulation. Given the versatility of TA in enhancing adhesion to tissue surface through poly‐OH moieties,^[^ ^23^ ^]^ the CPD‐TA nanoglue is slated for displaying multifarious activity including but not limited to delivering drug/growth factors, enabling its tracking, and generating visible light‐induced ROS for photoinduced self‐sterilization. Crucially, the CPD‐TA nanoglue was found to scavenge ROS as well. This implicates the uniqueness of this first‐ever reported nanoglue in delivering and absorbing ROS at different stages of wound healing as displayed through the chosen in vitro model of diabetic wound healing.

**Figure 1:**
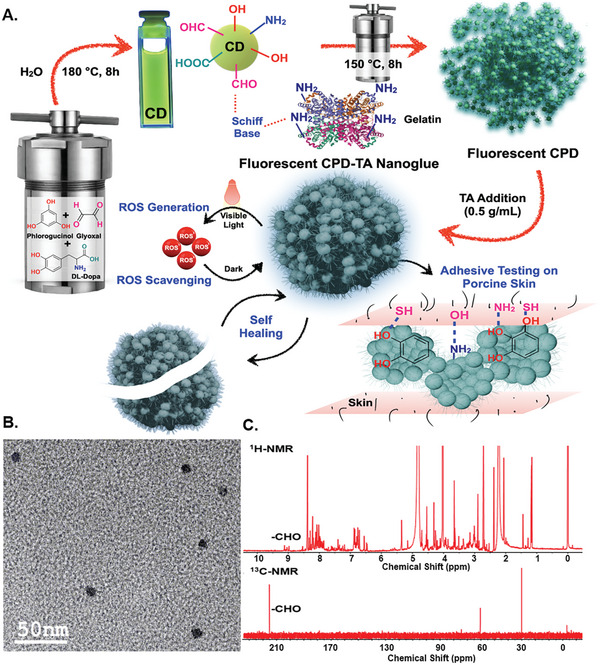
A) Design strategy for dual hydrothermal synthesis of CPD‐TA nanoglue for enhanced adhesion and ROS regulation under ambient conditions, B) TEM image of CD with an average diameter of less than 10 nm and C) 1H and 13C NMR of CD confirming ─CHO presence.

Prolonged hyperglycemia in diabetic patients disrupts cytokine secretion and fosters hyperinflammation, hypoxia, and persistent infections, exacerbating wound‐healing complications.^[^ ^24^, ^25^, ^26^ ^]^ Elevated glucose levels create a more conducive environment for bacterial growth than normal wounds, leading to the indiscriminate use of conventional antibiotics and the emergence of drug‐resistant bacterial strains.^[^ ^27^, ^28^ ^]^ While conventional healing and sterilization strategies include controlling glucose levels through enzymatic reactions^[^ ^29^ ^]^ and metal incorporation in adhesive hydrogels^[^ ^30^, ^31^, ^32^, ^33^, ^34^, ^35^, ^36^, ^37^, ^38^ ^]^ there is no report of any bioglue delivering the requisites at the wound site. Herein, insulin was chosen as a model growth factor for delivery from the nanoglue to the in vitro diabetic wound model since reportedly topical application of insulin has been found to accelerate healing.^[^ ^39^, ^40^ ^]^ Concurrently, ROS can effectively manage bacterial infections without resistance in the early stage but needs to be removed later for prosperous healing.^[^ ^41^, ^42^ ^]^ Understandably, in situ ROS generation and scavenging is an orthogonal aspect seldom taken care of simultaneously by a single agent. The same is achieved through the synergistic modulation of ROS generated from the CPD in the nanoglue with the added provision of stimuli‐free delivery of any drug/growth factor that hints toward a broader scope of personalized wound management in the future through a suitable nanoglue formulation (**Figure** **1**A).

## 2 Results and Discussions

### 2.1 Synthesis of CD, CPD, and CPD‐TA Nanoglue

The CPD‐TA nanoglue formation described here was achieved through a distinctive two‐step hydrothermal process where the first step was synthesizing the CD itself. CD was synthesized hydrothermally at 180 °C for 8 h using glyoxal (─CHO source), phloroglucinol (─OH source), and DL‐Dopa (─NH_2_ and ─OH source) to functionalize it with ─CHO, ─OH, and ─NH_2_ groups. The resultant quasi‐spherical CD obtained after purification was found to have an average size of less than 10 nm (Figure 1B) in transmission electron microscopy (TEM) imaging and lattice spacing of 0.22 nm calculated from the high‐resolution TEM (HRTEM image) (Figure S1, Supporting Information). Powder X‐ray diffraction analysis (pXRD) revealed the CD to be semi‐amorphous with two broad diffraction peaks indicative of heterogenous graphitic crystallinity, specifically observed at 31° and 42° aligning with the (002) and (100) planes, respectively (Figure S2, Supporting Information).

The major challenge was to retain the substrates' functional groups on the surface of CD during the pyrolysis for which the substrate stoichiometry, time, and temperature of the first hydrothermal step were determined through a combinatorial optimization exercise. Since the significant stake was to maximize the number of functional groups on the CD, the same was resolved by simple functional group analysis like ninhydrin test for ─NH_2_ and Schiff base assay for ─CHO groups of resultant CDs to pinpoint the substrate stoichiometries (Figures S3 and S4, Supporting Information). The presence of the functional groups on the surface of CD was further ascertained by Fourier transform infrared spectroscopy (FTIR) (Figure S5, Supporting Information). The distinct doublet peaks at 2860 and 2750 cm^−1^ and a sharp peak at 1725 cm^−1^ obtained in FTIR were attributed to the C─H and C═O stretching frequencies respectively testifying to successful retention of ─CHO groups on the CD surface. The broad peaks at 3200 cm^−1^ (O─H stretching) and 3500 cm^−1^ (N─H stretching) confirmed the presence of ─OH and ─NH_2_ groups on CD. The presence of ─CHO on the optimized CD was further ascertained through its characteristic strong peaks in ^1^H‐ nuclear magnetic resonance (^1^H‐ NMR) and ^13^C‐ NMR at 9.2 and 215 ppm, respectively (Figure 1C). The concentration of ─CHO groups on the CD was quantified through Schiff base assay revealing 4.02 mm ‐CHO in 10 µL CD (Figure S4B, Supporting Information) making it a potential crosslinker. May seem apparent, nevertheless, the retention of ─CHO on CD is challenging. Confirming their presence on purified CD surfaces and quantifying the same as demonstrated here is exceedingly rare in the literature.

The importance of the presence of ─CHO on the CD surface was comprehended through the formation of a dynamic Schiff base with ─NH_2_ groups of gelatins. Thus, the CD was conjugated with gelatin through appropriate surface functionality as a first cross‐linking level directly related to adhesion strength. However, the chemistry was further expanded by performing a second step of controlled hydrothermal pyrolysis to form CPD at 150 °C, which is 30 °C lower than that employed for CD formation. Subjected to prolonged pressure and temperature for 8 h, the CD‐gelatin crosslinked conjugate was transformed into CPD through partial carbonization. This process retained the desired functional groups of both CD and gelatin for tissue adhesion, as confirmed by FTIR spectra (Figure S6, Supporting Information). The concentration of CD and gelatin used for CPD formation was determined through optimization to achieve the best possible adhesive strength.

**Figure 2:**
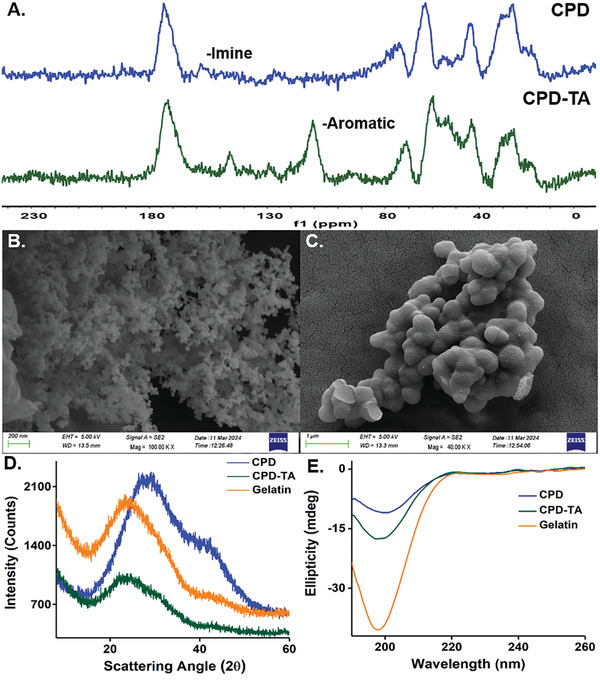
A) 13C CPMAS NMR data for CPD and CPD‐TA stating clear imine linkage and strong TA grafting, B) FESEM image demonstrating CPD with an average diameter of ≈50 nm, C) FESEM image of CPD‐TA nanoglue with size increment ≈50 nm, D) Comparative pXRD spectra show a drastic shift of peaks in CPD‐TA and E) Circular dichroism spectra show a broadening of the peak below the isoelliptical point.

To further enhance the potency and balance of adhesion and cohesion strength in the CPD, TA was introduced as a co‐component of the nanoglue through simple physical mixing.^[^ ^23^ ^]^ The final formulated CPD‐TA nanoglue showed a characteristic band at 1036 cm^−1^ in the FTIR spectra corresponding to the C═H vibration of the benzene ring near the phenolic ─OH groups of TA confirming the grafting of TA with the CPD (Figure S7, Supporting Information). The divulged amide A stretching at 3400 cm^−1^, O═H stretching at 3050 cm^−1^, amide II at 1570 cm^−1^ and broadening of amide I at 1650 cm^−1^ indicates abundant functional groups on the CPD‐TA surface while the gelatin backbone with important domains remains partially intact for skin adhesion. Solid‐state ^13^C cross‐polarization magic angle spinning (CPMAS) NMR data (**Figure** **2**A) provided additional evidence to support imine linkage formation in the CPD‐TA nanoglue by depicting broad peaks at 150 ppm. The grafting of TA on CPD was further justified with the emergence of new strong peaks at 65 and 110 ppm due to ─OH and aromaticity of TA respectively.

### 2.2 Morphological and Photophysical Characterization of CPD‐TA Nanoglue

Using field emission scanning electron microscopy (FESEM) we established the granular morphology of CPD with an average diameter of ≈50 nm (Figure 2B) which increased to ≈250 nm upon modification with TA in the optimized ratio of CPD‐TA (0.25 g TA with 2.6 g of CPD) (Figure 2C). While decreasing the stoichiometry of TA (0.15 g) reduced the size of the CPD‐TA nanograft, increasing TA concentration (0.4 g) further led to a highly diffused structure with reduced attributes in both cases (Figure S8, Supporting Information). In addition, the size of CPD (Figure S9, Supporting Information) and CPD‐TA (Figure S10, Supporting Information) was visualized from TEM images correlates well with FESEM findings. An interesting fact unfolds from selected area electron diffraction (SAED) patterns suggesting both CPD (Figure S9, Supporting Information) and CPD‐TA (Figure S10, Supporting Information) as a mixture of semi‐amorphous and crystallinity attributes that is different from that of CD (Figure S11, Supporting Information). As an obvious consequence, a gradual increment in size was observed in dynamic light scattering (DLS) studies where the average hydrodynamic diameter of the CD (≈52 nm) increased to ≈90 nm for CPD and further to ≈343 nm after TA grafting into the CPD (Figure S12, Supporting Information). Mean zeta potential predicts a positively charged CD with an average potential of +1.13 mV that shifted toward a negative value of −3.3 mV following its transformation into CPD with gelatin. This charge was partially masked after TA grafting on CPD resulting in the CPD‐TA value being −1.4 mV (Figure S13, Supporting Information).

Interestingly, a comparative pXRD study reveals a drastic shift of the dihedral angle peak at 28° in CPD to 23° in CPD‐TA comprehend to the structural changes in α‐helix of gelatin (Figure 2D). Broadening of the spectral peak below the isoelliptical point at 197 nm for CPD and CPD‐TA in circular dichroism spectra implies alterations in random coils of gelatin resulting from its Schiff base mediated crosslinking with CD and subsequent second step hydrothermal autoclaving leading to partial pyrolysis and further crosslinking.^[^ ^43^ ^]^ The distortion of gelatin secondary structure after CPD formation was reinforced through the observed shifting in far UV peaks at 221 nm indicating a mild weakening of the triple helix structure carried over after TA addition to CPD (Figure 2E). This observation is essential to confirm the preservation of the partial structural integrity of gelatin after hydrothermal treatment to enable it to maintain the fidelity of the polymeric nature of CPD and CPD‐TA.

### 2.3 Photophysical Characterization of CPD‐TA Nanoglue

**Figure 3:**
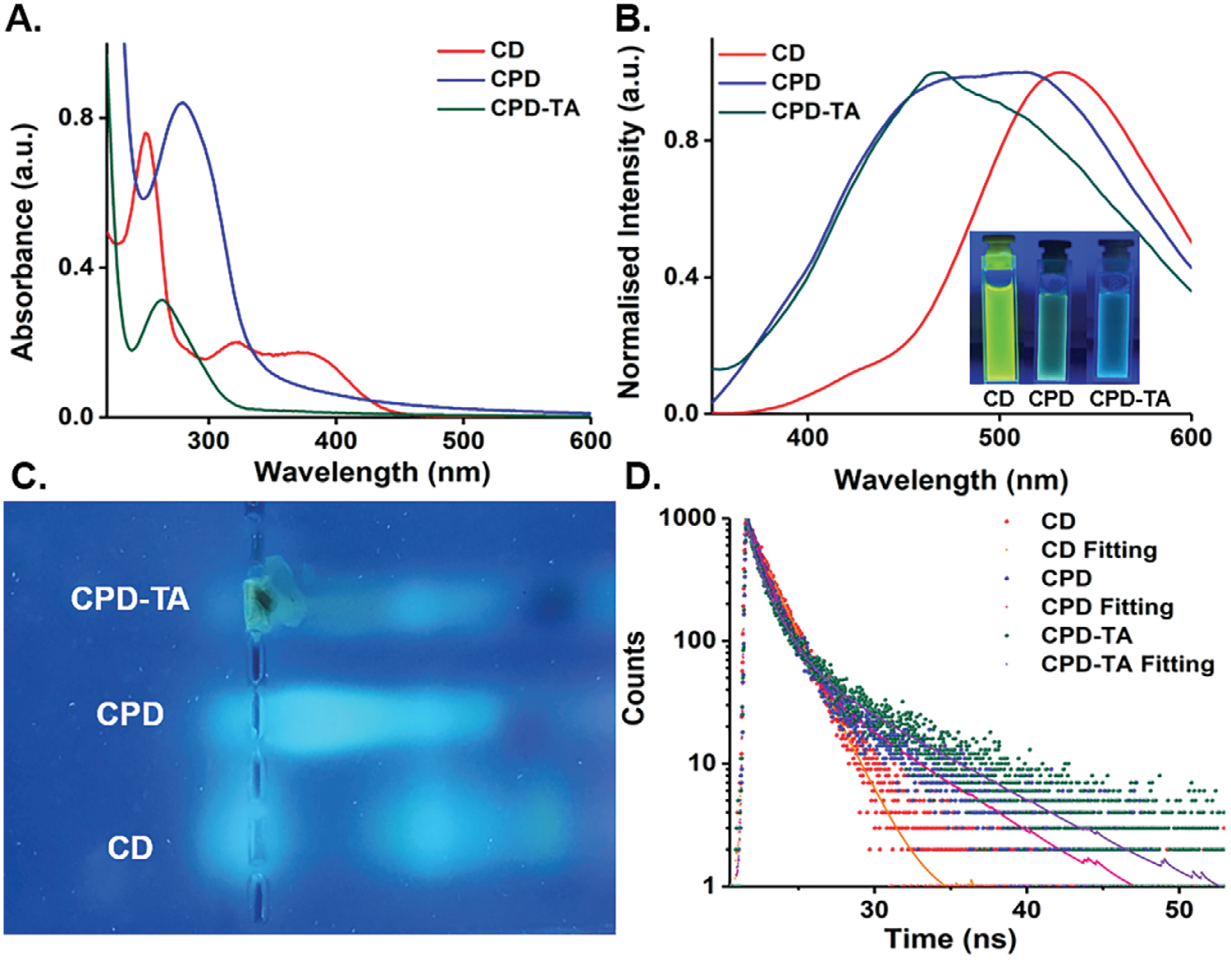
A) UV–visible spectra with blue‐shifted peaks in CPD and CPD‐TA nanoglue in comparison to CD, B) Emission spectra at 340 nm excitation with blue‐shifted peaks, C) 1.5% agarose gel validates separation between all the three having different fluorescence and D) Lifetime spectral analysis of CD, CPD, and CPD‐TA showed reduced lifetime in the latter.

CDs are characterized by their rich photophysics, which is no exception in this case. The photophysical aspects of our synthesized CD were found to be translated into CPD and ultimately to the CPD‐TA nanoglue in a predictable manner (**Figure** **3**A). A prominent peak at 251 and 320 nm alongside a broad shoulder ≈376 nm characterizes the absorption spectra of CD. While the peak at 251 nm typically indicates π–π^*^ transitions within the graphitic core, the broader absorption suggests n‐π^*^ transitions from surface functional groups, elucidating the complex surface chemistry inherent to CDs. Transitioning to CPDs, a red‐shifted absorption at 279 nm and a broadened spectrum at 348 nm were observed. This was attributable to the enhanced conjugation by imine bonds and interaction between the carbon core and the polymer matrix, thus modifying the electronic structure.^[^ ^13^, ^44^ ^]^ Upon further modification with TA to produce CPD‐TA, a blue absorption shift at 262 nm was noted concerning CPD resulting from altering the local electronic environment.

PL analysis revealed a notable change in emission when transitioning from green fluorescent CD to CPD and then to CPD‐TA via dual‐step hydrothermal synthesis (Figure S14, Supporting Information). The second step of controlled pyrolysis followed C═N bond formation between CD (─CHO) and gelatin (─NH_2_) resulting in CPD displaying bluish–green fluorescence at 340 nm excitation distinctly different from the reportedly faint blue fluorescence of gelatin‐based nanoparticle (Figure 3B).^[^ ^45^ ^]^ The grafting of TA further quenched and shifted the fluorescence to the Cynon region confirmed with emission at 465 nm with 340 nm excitation (Figure 3B). The intense PL of CD, CPD, and CPD‐TA enable a label‐free resolution in agarose gel as an indirect proof of the formation of CPD and CPD‐TA wherein distinct mobility of CD and CPD was observed along with extremely restricted mobility of the band corresponding to CPD‐TA due to enhanced crosslinking and grafting (Figure 3C). Average fluorescence lifetime measurements further complemented these findings, with CD exhibiting a relatively long lifetime of 3.55 ns, indicative of efficient radiative processes and minimal nonradiative decay pathways. In contrast, CPD showed a reduced lifetime of 1.38 ns, suggesting an increase in nonradiative decay mechanisms likely due to the polymer matrix's incorporation. This trend continued with CPD‐TA which presented an even shorter lifetime of 1.33 ns, pointing toward the influence of TA in promoting nonradiative decay processes or facilitating faster relaxation of excited states (Figure 3D; Table S1, Supporting Information). The difference in PL of the CD, CPD, and CPD‐TA nanoglue can be advantageously applied to determine the integrity of the nanoglue after its application wherein the emission color from a momentary UV‐torch irradiation can tell whether the nanoglue is disintegrating with time or not.

### 2.4 Evaluation of Dual Regulation of ROS Generation and Scavenging by CPD‐TA Nanoglue

**Figure 4:**
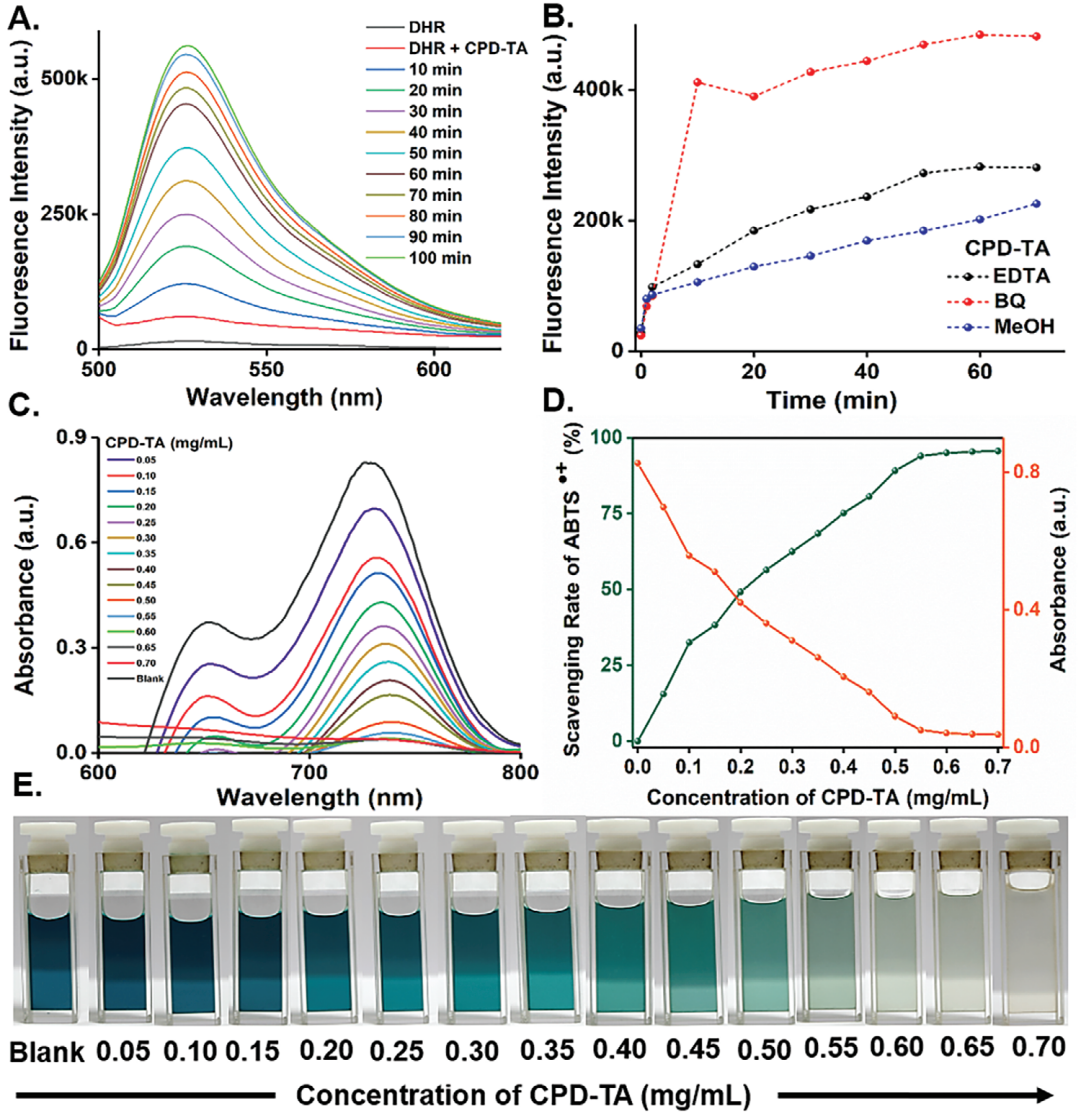
A) Fluorescence spectra at 480 nm excitation for CPD‐TA nanoglue in the presence of DHR123, B) Comparison w.r.t fluorescence intensity for trapping free radical species for CPD‐TA, C) UV–visible spectra of ABTS assay with varying concentrations of CPD‐TA (mg mL^−1^), D) Scavenging rate of ABTS**^˙+^** versus concentration of CPD‐TA relation with absorbance and E) Digital images of ABTS assay.

Photoactivated CDs are known to generate ROS by various mechanisms owing to their multiple electronic states and surface defects. We observed the retention of the ROS generation ability of the CD even after their transformation into CPD and CPD‐TA just by visible light irradiation. ROS generation capacity of CD (Figure S15, Supporting Information), CPD (Figure S16, Supporting Information), and CPD‐TA (**Figure** **4**A; Figure S17, Supporting Information) were semi quantitatively evaluated under visible light irradiation (white LED, 23 W) by converting non‐fluorescent Dihydrorhodamine 123 (DHR123) to green fluorescent Rhodamine 123. We registered a negligible change in the fluorescence of DHR 123 in the presence of CD, CPD, and CPD‐TA in the absence of the light source, stating inhibition of ROS generation and hence minimalistic dark activity (Figures S15B, S16B, and S17, Supporting Information). To dig further into the plausible mechanism of ROS, trapping of the generated free radicals was done using well‐known trapping reagents. The hydroxyl (**^.^** OH) was trapped using methanol (MeOH), Na_2_‐EDTA trapped surface generated hole (h**^+^**) while for superoxide (O**^2−^**) trapping parabenzoquinone (BQ) was used where a decrease in fluorescence intensity (emission: 525 nm) in the presence of a particular radical confirms its presence. A maximum reduction in fluorescence intensity was observed for CD (Figure S18, Supporting Information) and CPD (Figure S19, Supporting Information) using Na_2_‐EDTA suggesting more surface‐generated h**^+^** presence with unique recombination phenomena responsible for these trapped ROS. However, CPD‐TA nanoglue showed a decrease in fluorescence in MeOH followed by Na_2_‐EDTA (Figure S20, Supporting Information) suggesting the dominance of OH radical and surface‐generated h**^+^** in CPD‐TA (Figure 4B) resulting from the TA grafting with CPD.

In an exciting and concurrent finding, we observed the ROS scavenging ability of CD that was significantly amplified in CPD and CPD‐TA nanoglue in the absence of light. The crossover between oxidant–antioxidant studies was considered with various control experiments to establish the rare phenomena of simultaneous ROS generation and scavenging, the latter being instrumental due to abundant OH groups in CPD and even more in CPD‐TA. Unoxidized catechol groups or polyphenols are well known for their free radical scavenging activity in addition to their tissue‐adhesion attributes. The ROS scavenging by the nanoglue is a direct consequence of functional group retention of the substrates throughout their transformation (CD→CPD→CPD‐TA) during two‐stage controlled pyrolysis. The multitude of scavenging activity of CD (Figure S21, Supporting Information), CPD (Figure S22, Supporting Information), and CPD‐TA nanoglue (Figure 4C) was demarcated to assess the total antioxidant activity using ABTS assay. The chromogenic assay decolorizes 98.5% ABTS**^˙+^** to ABTS with 1.5 mg mL^−1^ for CD, 2 mg mL^−1^ for CPD, and 0.7 mg mL^−1^ for CPD‐TA (Figure 4D,E) signifying enhanced scavenging activity by CPD‐TA owing to abundant ─OH functionality. It is worth mentioning here that ROS and scavenging are required at different stages of wound healing. To the best of our knowledge, these findings are a unique contextual outcome wherein the nanoglue formulation is simultaneously able to generate ROS and scavenge the same from the microenvironment under ambient conditions as per the requirement of the wound healing process.

### 2.5 Thermal, Mechanical, and Self‐Healing Properties of CPD‐TA Nanoglue

The thermal stability of the developed CPD‐TA was evaluated through TGA analysis by adjusting different amounts of TA (0.15, 0.25, and 0.40 g) to CPD (2.6 g) toward CPD‐TA formulation. The study revealed a three‐phase thermal decomposition pattern for all samples, mirroring pure gelatin but initiating at elevated temperatures. Specifically, the onset temperature for a 5% weight loss in gelatin was recorded at ≈82 °C attributed to moisture evaporation. In contrast, the CPD exhibited this initial weight loss at a higher temperature of 154 °C. The CPD‐TA nanoglue demonstrated excellent thermal stability with the onset temperatures of 223.5 °C for 0.25 g TA which was optimum compared to reduced onset temperature for other combinations (180 °C for 0.15 g and 215 °C for 0.40 g TA). This progression emphasizes the enhanced thermal resilience imparted by incorporating TA into the CPD matrix (Figure S23, Supporting Information).

**Figure 5:**
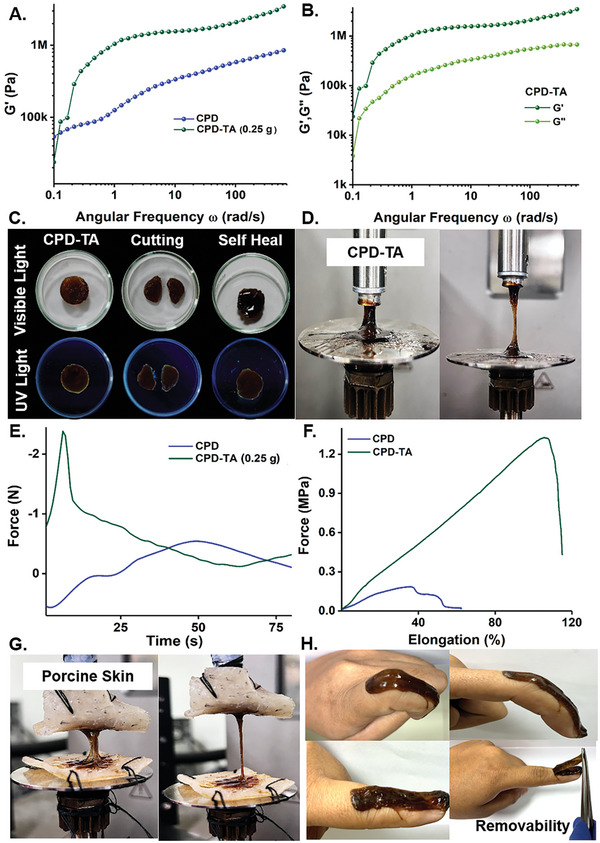
A) Storage modulus increase after incorporation of TA in CPD matrix confirmed with a frequency sweep, B) Bulk rheological parameters of stiffness inferred *G*′ dominance over *G*″ across a wide range of oscillatory shear frequencies, C) Instant self‐healing of CPD‐TA visualized under regular and UV light, D) Digital images of nanoglue on steel parallel plates maintaining fibrillation even after 80 mm distance, E) Probe tack test validating superior fibrillations and cavitation in optimized CPD‐TA nanoglue, F) Lap shear test conducted on porcine skin with CPD‐TA, G) Stretchability test on porcine skin displaying strong fibrillation even after 80 mm distance and H) Images of adhesive patch on human skin adapting finger movement along with easy removability.

An ideal adhesive protects chronic wounds from secondary damage while maintaining structural integrity and appropriate elasticity. The effect of interfacial interaction of the CPD and its impact on mechanical properties as a function of TA content in the nanoglue was evaluated. The optimum CPD‐TA formulation (0.25 g TA with 2.6 g CPD) displayed the highest G′ of 2.9 MPa suggesting remarkable cohesion and interfacial interaction with superior elastic characteristics (**Figure** **5**A; Figure S24, Supporting Information). The macroscopic properties characterized by bulk rheological parameters of stiffness storage modulus (*G*′) and loss modulus (*G*″) inferred *G*′ dominance over *G*″ across a wide range of oscillatory shear frequencies signifying the stable and elastic nature of the nanoglue (Figure 5B). This elastic dominance, coupled with a lower loss tangent for the optimized CPD‐TA points to the nanoglue‐enhanced cohesive nature and reduced polymer chain mobility with TA grafting into CPD (Figure S25, Supporting Information).

The formed CPD‐TA nanoglue is adaptable to mold into irregular shapes and accommodate skin movement without being exposed to external stimuli. These reversible bonding mechanisms are primarily attributed to the rescindable interactions among the functional groups like aldehyde (─CHO), amine (─NH_2_), and hydroxyl (─OH) present in the CPD‐TA. Majorly, hydrogen bonding between the ─OH groups and other available functional groups contributes to the self‐healing capability. This combination of interactions allows the immediate re‐attachment of the nanoglue upon being cut and re‐aligned instantly and concurrently the self‐healed nanoglue manifested stretchability to an optimum extent (Figure 5C; Figure S26, Supporting Information). To maintain a healing and moist environment for wounds, the swelling capacity of the nanoglue matrix is vital for encapsulation, which was determined in the present case over 5 days in phosphate‐buffered saline (PBS) at room temperature.^[^ ^46^ ^]^ The swelling curve of CPD shows a time‐dependent increase in which it swelled excessively (40%) and disintegrated relatively quickly within 12 h. However, CPD‐TA nanoglue showed a low to moderate weight increase (≈27.7%) in the initial 8 h before achieving an equilibrium state. Following a similar stability trend, CPD starts to disintegrate within 12 h while in contrast, higher crosslinking in CPD‐TA makes it stable in PBS beyond 4 days (Figure S27, Supporting Information).

### 2.6 Adhesion and Cohesion Interconnection in CPD‐TA Nanoglue

The tackiness of the nanoglue was perceptible upon direct contact and was quantitatively and visually assessed through a probe tack test on steel parallel plates maintaining fibrillation even after an 80 mm distance (Figure 5D). The optimized CPD‐TA (TA: 0.25 g) nanoglue showcased maximum resistance to separation during the debonding process with the highest peak force of 2.36 N (Figure 5E). This indicates higher energy dissipation with superior cavitation and fibrillation characteristics in the optimized formulation of the nanoglue compared to CPD and non‐optimum ratios of CPD‐TA (Figure S28, Supporting Information). An adhesion test was also conducted with porcine skin to emphasize the functional groups' synergistic effect of CPD‐TA. That includes polyphenolic moieties to ensure robust adherence through a network of supramolecular and covalent interactions with the skin's natural functional groups. The effectiveness of the nanoglue was quantitatively confirmed through improved adhesion strength and its co‐dependency on cohesion in porcine skin tissue by lap sheer test (Figure 5F; S29, Supporting Information). CPD revealed minimal adhesion to the porcine skin of 0.17 MPa whereas in the optimized CPD‐TA (0.25 g) nanoglue adhesive strength increased significantly reaching 1.32 MPa. We inferred higher strength in the latter due to adequate polyphenolic moieties making the nanoglue a superior sealant. The stretchability test conducted on porcine skin (Figure 5G) demonstrates that the nanoglue can maintain strong fibrillation up to 80 mm even under external force. One noteworthy takeaway is the exceptional strength of the optimized nanoglue when compared to other bioadhesives reported in current literature, which typically exhibit strengths ranging from 0.001–0.57 MPa (Table S2, Supporting Information). The nanoglue not only adheres well to the fingers but also adapts well to finger movement along with easy removability and stretchability (Figure 5H). Moreover, nanoglue adheres well to various substances like glass, plastic, paper, rubber, and gloves, as visually displayed in Figure S30 (Supporting Information).

### 2.7 Insulin Loading and Release Kinetics into CPD‐TA Nanoglue

**Figure 6:**
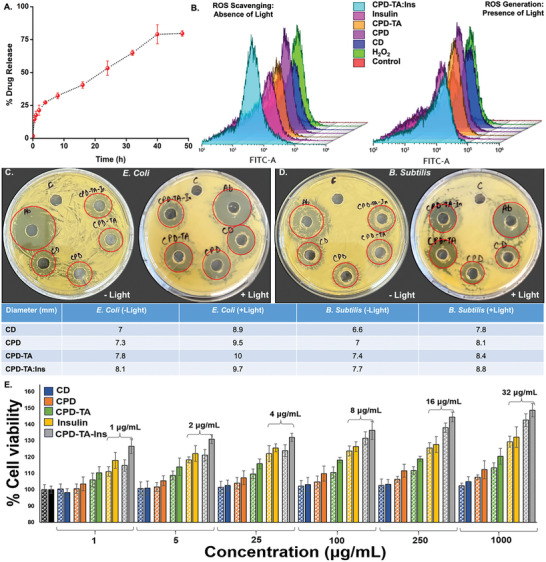
A) % Insulin release kinetics of CPD‐TA: Ins over the time period of 48 h, B) DCFDA assay demonstrating ROS scavenging and generating efficacy of CPD‐TA: Ins nanoglue in the presence and absence of light, C) Zone of inhibition method with the antibacterial activity of *E. coli* in the presence and absence of light, D) Zone of inhibition method with the antibacterial activity of *B. subtilis* in the presence and absence of light, and E) % Cell viability tested on HEKa cells under light showing CPD‐TA: Ins to promote cell division and growth in normal wounds (solid bars) and diabetic wounds (patterned bars) for 24 h. All the statistical significance of data is considerable with *p* < 0.05.

The synthesized nanoglue was evaluated for its drug loading and release kinetics. The loading capacity of insulin‐loaded CPD‐TA (CPD‐TA: Ins) was determined to be 29.9 ± 0.8 µg mL^−1^, with a loading efficiency of 93.50 ± 2.84%. Over 48 h, 79.62 ± 2.04% of the drug was released from the nanoglue, demonstrating its efficiency as a drug delivery system, as shown in **Figure** **6**A. An initial burst release was observed for 1 h followed by sustained drug release over the remaining time, with the majority of the drug being released within 48 h (Figure S31, Supporting Information).

### 2.8 Intercellular ROS Measurement for CPD‐TA Nanoglue

Flow cytometry was performed to examine the ROS‐generating and scavenging activity of synthesized nanoformulations. Briefly, the DCFDA probe which is non‐fluorescent exhibits green fluorescence when comes in contact with ROS as determined by measuring the fluorescence at 572 nm. Initial analysis was conducted on control cells and cells containing only the DCFDA probe taken as control. The ROS activity of CPD‐TA was then determined by measuring fluorescence in cells treated with H_2_O_2_, followed by cells treated with CD, CPD, CPD‐TA, insulin, and CPD‐TA: Ins. The results show prominent scavenging of H_2_O_2_‐induced ROS in the presence of all formulations. The percentage increase in ROS after treatment with H_2_O_2_ was found to be 16.10% which decreases significantly to 1.98% in CPD‐TA: Ins (maximum) in the absence of light indicating their ROS scavenging potential (Figure 6B). In the presence of light, the cells showed enhanced ROS generation with the maximum observed in cells treated with CPD‐TA (38.06%) which coincides well with the DHR123 studies and ABTS assay (Figure 6B). A comparative table of ROS measurement both in the presence and absence of light is given in Table S3 (Supporting Information).

### 2.9 Photoactivated Bacterial Annihilation and Cell Viability of CPD‐TA Nanoglue

The minimum inhibitory concentration (MIC) values were determined against Gram‐negative (*E. coli*) and Gram‐positive bacteria (*B. subtilis*) and came out as 100 and 200 µg mL^−1^ respectively, showing more lethality of the nanoglue for the former. For valid comparisons in both bacterial strains, the agar well diffusion method under light exposure for 1 h as well as dark (Figure 6C,D) was considered. For *B. subtilis*, the zone of inhibition without light is maximum for CPD‐TA: Ins with a value of ≈7.7 mm which further increased to ≈8.8 mm under visible light exposure. Even better results were obtained for *E. coli* where CPD‐TA: Ins again have a maximum zone of inhibition with ≈8.1 mm which further amplified to ≈9.7 mm under visible light irradiation. Under dark conditions, for all other control samples viz. CD, CPD, and CPD‐TA the zone of inhibition increased for both *E. coli* and *B. subtilis* while going from dark to irradiated conditions. The noteworthy dark activity of nanoglue is because of imine functionalization which has proven antibacterial action.^[^ ^13^ ^]^ The strong light‐induced antibacterial activity is the result of the synergistic effect of the multifunctional groups' domain that generates surface‐activated holes in CPD‐TA nanoglue responsible for effective bacterial annihilation leading to self‐sterilization of the wound without externally added antibiotics for potent recovery and prosperous healing.

Cell viability analysis was performed using HEKa cells through MTT assay to monitor the biocompatibility of the synthesized CPD‐TA formulations that can generate ROS. Cell viability was evaluated in the presence of six different concentrations each of CD, CPD, CPD‐TA, insulin, and CPD‐TA: Ins in both normal as well as diabetic conditions (both in the absence and presence of white light) wherein the cell viability of untreated cells was taken as control (100%). All the samples showed excellent biocompatibility, however, CPD‐TA was found to be the most biocompatible under both dark and light conditions in normal as well as diabetic wound conditions (Figures S32, S33, and Tables S4, S5, Supporting Information). Most encouragingly, a significant synergistic effect was observed in the presence of insulin using CPD‐TA: Ins (1000 µg mL^−1^ CPD‐TA and 32 µg mL^−1^ insulin) that amplified cell viability to 149.39 ± 1.98% and 144.16 ± 3.21% in normal and diabetic wound model respectively for 24 h. We have monitored CPD‐TA nanoglue toxicity for an extended period and found that none of the synthesized formulations is toxic to skin cell line (HEKa) in normal (Figure S33 and Table S4, Supporting Information) and diabetic conditions (Table S5, Supporting Information) even after 48 and 72 h. In addition, the CPD‐TA: Ins (1000 µg mL^−1^ CPD‐TA and 32 µg mL^−1^ insulin) acts as a cell growth promoter with an increase in cell viability to 209.75 ± 5.19 and 185.39 ± 2.02% under normal and diabetic conditions after 72 h of treatment.

In summary, it is established beyond doubt that CPD‐TA is nontoxic to cells and that the addition of growth factors increases cell viability (Figures S32, S33, and Table S4, Supporting Information). The proportional increase of cell viability with an incremental concentration of CPD‐TA: Ins only indicates that the formulation promotes cell division and growth and thus can be used as potent material for wound healing in normal conditions. Under diabetic conditions, a similar trend was observed (Figures S32, S33, and Table S5, Supporting Information) except the increase in cell viability is slightly lower than that under normal conditions due to the apparent reason for slower growth and cell division rate in a diabetic microenvironment.

The effect of ROS was thoroughly studied in both the wound models where cells were irradiated for 1 h using white LED (23 W). Augmented cell viability was observed for CPD‐TA: Ins (148.74 ± 4.14%) as against pristine CPD‐TA (120.48 ± 4.93%) (Figure 6E; Table S6, Supporting Information) in normal wounds. A similar trend was detected for diabetic wounds where cell viability in the presence of CPD‐TA (113.43 ± 3.11%) increased after insulin loading (CPD‐TA: Ins; 142.64 ± 3.85%) emphasizing no effect of white light incubation on the viability of human cells (Figure 6E; Table S7, Supporting Information). To find the statistical significance of data, p values were calculated for % variation cell viability in diabetic and normal conditions, and the comparative data is shown in Tables S8–S11 (Supporting Information). Conviction in the efficacy of CPD‐TA nanoglue in promoting cell division and growth is firmly established accentuating the fact that the quantum of ROS generated from CPD‐TA may be lethal to bacteria. Still, the dosage is too small to affect human cells adversely.

### 2.10 HEKa Cell Migration Assay in Normal and Diabetic Wound Conditions for CPD‐TA Nanoglue

**Figure 7:**
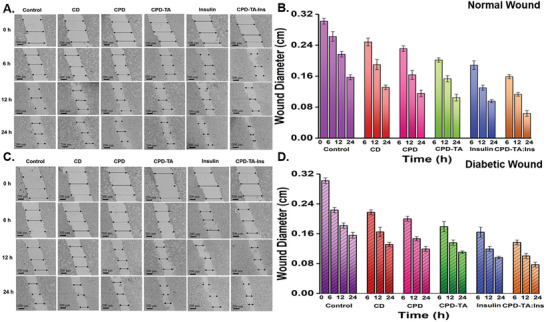
A) HEKa cell migration with different formulations after time intervals of 0, 6, 12, and 24 h in normal wound. B) Wound diameter (cm) change in all the formulations for normal wound, C) HEKa cell migration with different formulations after time intervals of 0, 6, 12, and 24 h in the diabetic wound, and D) Wound diameter (cm) changes in all the formulations for the diabetic wound. The statistical significance of data is considered when p < 0.05.

Following up on the MTT assay, CPD‐TA nanoglue was tested for its potential role in normal and diabetic wound healing on HEKa cells by treating them with a fixed concentration (1000 µg mL^−1^) of CPD‐TA, insulin, and CPD‐TA: Ins (1000 µg mL^−1^ of CPD‐TA and 32 µg mL^−1^ of insulin) along with CD and CPD as controls. They exhibited excellent healing abilities in both conditions but better impacted normal wounds than diabetic ones. A significant increase in cell division with time eventually leads to enhanced migration rates when monitored after specific intervals. Initially, the % change in wound diameter is measured in HEKa cells taken as control (without any treatment) after 0, 6, 12, and 24 h in both normal (**Figure** **7**A,B) and diabetic wounds (Figure 7C,D). A comparative data table of changes in wound migration in normal and diabetic wounds over time is shown in Table S12 (Supporting Information).

The initial testing was done on CPD‐TA for normal and diabetic wounds where the % change in scratch diameter of cells was found to be 33.42 ± 0.80% and 29.14 ± 0.36% after 24 h. The influence of insulin was studied where the maximum potency of CPD‐TA: Ins increased to be perceived at 59.41 ± 0.75% and 50.53 ± 0.66% after 24 h for normal and diabetic wounds respectively. To find the statistical significance of data, p values were calculated for scratch assay in diabetic and normal wound conditions, and the comparative data is shown in Table S13 (Supporting Information). Improved healing outcomes are attributed to the enhanced functionality of nanoglue, which features various groups like carbonyl, amine, carboxy, and hydroxyl. These groups play vital roles in aiding biochemical processes including but not limited to cell signaling, immune response, and supramolecular interactions (including hydrogen bonding through hydroxyl groups) facilitating interactions between molecules involved in the wound healing process. This indicates the enormous potential of the prepared CPD‐TA nanoformulation in the healing of normal and diabetic wounds, along with the excellent adhesion and cohesion strengths expected from a bioglue.

To summarize, DL‐Dopa (catechols) and phloroglucinol are important molecules in nature related to adhesion in mussels and algae.^[^ ^47^, ^48^ ^]^ However, their biomimetics necessitate stronger adhesion for bioadhesive applications, typically achieved through aldehyde‐based crosslinking, although with proven toxicological implications.^[^ ^49^ ^]^ This prompted us to co‐nanotize Dopa, phloroglucinol, and glutaraldehyde in our synthesis of CD to encompass three main reasons concerning nanoglue. First, the biomimetic chemical functional groups of the substrates are used for enhanced adhesion–cohesion and second, the possibility of release of free aldehyde is shunted out. Lastly, novel properties of the resultant nanoparticle add value to the product. The CD was well characterized by FTIR and NMR to ascertain surface functional groups, UV, PL, and lifetime spectroscopy for photophysics, and TEM, DLS, SEM, and pXRD for morphological findings. The CD was conjugated through Schiff base formation with gelatin, a nontoxic, edible protein with known weak adhesion properties. Subsequently, second‐stage pyrolysis yielded CPD as a biomimetic tool to create a nanoglue. Worth mentioning here, the mere addition of the three organic precursors during the second stage of pyrolysis co‐incubating with gelatin instead of the CD does not result in a bioglue with desirable attributes justifying nanotization concept (Figures S34–S36, Supporting Information). CPD was grafted with TA, a natural constituent of many plants^[^ ^50^ ^]^ to obtain CPD‐TA nanoglue and both were characterized with TEM, FESEM, DLS, and Zeta potential measurement with TA grafting established by CPMAS studies. The delicate balance of adhesion–cohesion was established by evaluating physico–mechanical properties through rheology, probe tack, and lap shear tests. Having achieved the first two goals of presenting biomimetic functionalities in a single nano‐ensemble for enhanced adhesion and eliminating toxicity from free aldehyde and looked upon the CPD‐TA nanoglue to extract novel properties generally not found in commercial ones. Indeed, successfully demonstrated the ability of CPD‐TA to be tracked through its PL properties and ability to deliver a drug/growth factor cargo (insulin). Values obtained for the combination index (CI) of CPD‐TA and insulin upheld excellent healing irrespective of their concentrations in a given nanoglue formulation, indicating synergism for enhanced activity. This coheres to results obtained from scratch assay for cell migration studies, antibacterial activity, and MTT assay for cell viability (Table S14, Supporting Information) outlining the multitasking ability of the nanoglue. Most interestingly, visible light‐induced ROS generation for self‐sterilization and simultaneous ROS scavenging in the dark for accelerated healing are novelties also obtained from the nanoglue. Overall, the scientific rigor embraces ≈20 analytical techniques to firmly establish the claim of a superior nanoglue with outstanding reproducibility allured for biosealant applications.

## 3 Conclusion

We are witnessing significant research efforts in bioadhesive/biosealant mainly to achieve three critical concurrent aspects viz. non‐toxicity, superior adhesion–cohesion, and localized drug/growth factor delivery for better wound management. Unfortunately, there are no clear winners as constituents of biosealants among synthetic polymer‐based systems and natural biopolymers. While synthetic polymers deliver optimal adhesion, they may induce increased chronic inflammation or higher cytotoxicity from potentially toxic degradation products and the latter suffers from below‐par adhesion–cohesion. Thus, hypothesizing a completely nontoxic biosealant from a natural polymer displaying excellent adhesion–cohesion at par with polymeric counterparts while the concurrent provision to deliver drug/growth factor for expedited healing is rousing to our research efforts. Inspired by nature that uses simple chemical functional groups of a handfuls of compounds to affect tissue adhesion, we ventured into the nanotization of three chosen ones (dopamine, phloroglucinol, and glutaraldehyde). Through a controlled pyrolysis process, a single nano‐entity (CD) was created that retains the functional groups like ─OH, ─NH_2,_ and crucially, ─CHO on the surface inherent to the substrates. This CD was made to act as a crosslinking core with an edible protein (biopolymer), gelatin through Schiff base formation. In a novel intervention, we went one step further with a second pyrolysis step with the CD‐gelatin conjugate that completely randomized the crosslinking with the formation of CPD. The exquisiteness of the controlled pyrolysis is demonstrated not only through the retention of the functional groups of both CD and gelatin in CPD that are key to superior adhesion–cohesion but also through the retention of CD PL. A nanoformulation was developed with CPD grafting with TA that left us with a non‐cytotoxic nanoglue. This nanoglue features a porous network conducive for loading drug/growth factor, PL for optical tracking, and excellent adhesion–cohesion defying the notion that biopolymers lack those last attributes. Having ticked all the boxes for a desirable biosealant, an additional feature of nanoglue was discovered which to the best of our knowledge is reported herein for the first time. The CPD‐TA nanoglue could be excited with a mere 23 W LED to generate ROS as a function of irradiation time, enough to kill Gram‐positive and Gram‐negative bacteria efficiently without harming human cells. The nanoglue could simultaneously scavenge ROS in the dark, otherwise achieved with typical antioxidants like ascorbic acid addition in traditional formulations. This aligns with the requirement of expedited wound healing where ROS flux is advantageous at the initial stages while its elimination is warranted at later stages. The all‐inclusive intelligent nano platform was poised to perform in an in vitro diabetic wound model with remarkable success. We believe this newly found double‐pyrolytic and subsequent grafting methodology could pave the way not only for superior reinforcement of damaged/cut tissues but also for comprehensive normal and specialized wound management using specific drug/growth factors as deemed relevant under certain disease conditions.

## 4 Experimental Section

### Synthesis of CD

The substrates of the CD were chosen to present functional groups like ─CHO, ─NH_2_, and ─OH on the surface. The synthesis involved a mixture consisting of glyoxal (3 mmol), phloroglucinol (0.5 mmol), and DL‐Dopa (1 mmol) dissolved in 7 mL of deionized water. This mixture was then sealed in a Teflon‐lined autoclave chamber and placed in a hot air oven at 180 °C for 8 h, forming a dark yellow supernatant. Subsequently, a yellow–green fluorescent CD was obtained after syringe filtration that eliminates large aggregates. Dialysis was employed to purify further the synthesized CD involving a membrane with a 2 kDa molecular weight cutoff (MWCO) with occasional changes of water for 48 h. This purification removes unreacted substrates and molecular fluorophores, yielding a refined CD product. The aqueous dispersed purified CD (aliquot 20 mg mL^−1^) was then stored at 4 °C for subsequent use.

### Synthesis of CPD and Modification with TA

A 30% (w/v) gelatin solution was prepared by dissolving gelatin in 4 mL water. To this solution, 0.5 mL of previously synthesized CD was added and stirred at room temperature for 5 mins. The resulting mixture underwent a second autoclaving process at 150 °C for 8 h to form the desired adhesive CPD. As an optimization exercise to obtain maximum adhesion, the CPD volume was reduced to 2 mL (2.6 g) and systematically modified by adding varying ratios of TA solution (0.5 g mL^−1^) in aliquots of 300 µL (0.15 g), 500 µL (0.25 g), and 800 µL (0.40 g) to separate 2 mL portions of the CPD, maintaining a constant pH of 8. This procedure facilitated the creation of distinct CPD‐to‐TA ratios, thereby facilitating a comprehensive investigation into the adhesive characteristics of the CPD about TA concentration.

### Light‐Induced ROS Generation

CD (1 mg mL^−1^), CPD (10 mg mL^−1^), and CPD‐TA (10 mg mL^−1^) were illuminated using a 23 W white LED source positioned 15 cm away for 1 h. ROS production under photoexcitation was evaluated by converting non‐fluorescent Dihydrorhodamine 123 (DHR 123, 1 µL of a 1 nm solution) into fluorescent rhodamine 123, serving as an indicator of ROS generation. This conversion was quantitatively measured using steady‐state fluorescence spectroscopy. At an excitation wavelength of 480 nm, emission spectra were recorded in the range of 490 to 650 nm to monitor the fluorescence intensity of rhodamine 123. This allowed for direct monitoring of ROS generation within the system. To trap free radicals, a solution containing 1 m MeOH, Na2‐EDTA, and BQ was prepared. This solution was added at an aliquot strength of 10 µL mL^−1^ to the final solution, and the amount of ROS generation was assessed using DHR 123, as described earlier.

### ABTS Assay for ROS Scavenging Activity of CD, CPD, and CPD‐TA Nanoglue

For the preparation of ABTS radical solution, 7 mm ABTS stock solution (500 µL) and 2.45 mm ammonium persulphate (APS, 500 µL) were mixed in 1:1 proportion and stored in the dark at room temperature for 2 h. ABTS loses an electron and forms ABTS**^˙+^** wherein the radical absorbs at 743 nm and gives a blue–green color. 300 µL of this ABTS radical solution was diluted with 200 µL of water to be designated as the blank solution. The CD (1 mg mL^−1^), CPD (10 mg), and CPD‐TA (10 mg) were separated in titration mode (20 to 200 µL) and absorbance was recorded to evaluate the inhibition percentage of the radical scavenging activity as calculated from the following equation:

(1) $$Inhibition\left(\%\right)=\left(A_{\mathrm{blank}}-A_{\mathrm{sample}}/A_{\mathrm{blank}}\right)100$$

where *A*_blank_ is the absorbance of ABTS (blank solution) and *A*_sample_ is the absorbance of ABTS after incubation with CD, CPD, or CPA‐TA at 743 nm.

### Swelling Index Ratio of CPD and CPD‐TA Nanoglue

The swelling characteristics of the nanoglue were investigated using a modified gravimetric method. Initially, 100 mg of the CPD and CPD‐TA was exposed to conditions simulating the physiological environment by incubating it in phosphate‐buffered saline (PBS) at a constant temperature of 37 °C with gentle agitation. The mass of the bioadhesive was recorded at predefined intervals to track its absorption capacity over time. The swelling ratio was quantitatively evaluated by comparing the mass before swelling (W_0_) with the mass after swelling at a given time point (*W*_t_), calculated using the equation:

(2) $$SwellingRatio(\%)=[(W_{t}-W_{0})/W_{0}]\times 100$$

### Insulin Loading and its Release Kinetics

Insulin was chosen as drug/growth factor cargo to encapsulate in CPD‐TA nanoglue. To monitor the drug loading (32 µg mL^−1^) in the nanoglue, 10 mL of the formulated CPD‐TA: Ins was taken and centrifuged at 6000 rpm for 15 min. The supernatant and pellet were then collected to quantify the bound and free drug, thereby determining the loading capacity of the formulations. Next, the drug release pattern was evaluated to assess the amount of insulin released from the nanoglue and determine its drug delivery efficiency. 10 mL of the nanoglue was placed in a dialysis membrane and suspended in 250 mL of PBS buffer at pH 7.4, maintained at 37 °C under slow stirring conditions (250–300 rpm). Samples (2 mL) were withdrawn at specific intervals and replaced with fresh PBS to maintain constant volume over 48 h. Subsequently, 200 µL of each sample was added to a 96‐well plate, followed by the addition of 10 µL of the Bradford's reagent. After 10 min incubation, absorbance values were measured at 595 nm and plotted using BSA standard curves to analyze the trend of drug release. The final insulin concentration was varied from 1, 2, 4, 8, 16, and 32 µg mL^−1^ with a respective concurrent concentration of CPD‐TA ranging from 1, 5, 25, 100, 250, and 1000 µg mL^−1^. The optimum activity was individually established for antibacterial, MTT, and cell migration.

### Intercellular Detection of ROS Using DCFDA Assay

ROS generation was determined by flow cytometry using DCFDA staining. The HEKa cells were cultured in DMEM media and initially treated with a fixed concentration of H_2_O_2_ (40 µm) for 1 h. Thereafter, the media containing H_2_O_2_ was discarded, and cells were treated with synthesized formulations including CD, CPD, CPD‐TA, insulin, and CPD‐TA: Ins having a final concentration of (250 µg mL^−1^) for 12 h in fresh DMEM media. The entire experiment was conducted in two steps, both in the presence (6 h) and absence of light to evaluate the impact of light on ROS generation and scavenging activity. After treatment, the cells were detached using trypsin‐EDTA solution suspended in 0.5 mL of PBS and incubated with DCFDA (10 µm) for 20 min before the flow cytometric analysis.

### Antimicrobial Activity of CPD‐TA Nanoglue

All the prepared samples of CPD and CPD‐TA having different concentrations ranging from 6.25, 12.5, 25, 50, 100, 200, and 400 µg mL^−1^ were tried. Further, for the specified range of CPD‐TA, insulin drugs (ranging from 1, 2, 4, 8, 16, and 32 µg mL^−1^) named insulin‐loaded CPD‐TA (CPD‐TA: Ins) were loaded. The MIC value against the antimicrobial activity of CPD, CPD‐TA, and CPD‐TA: Ins were tested using a standard protocol. The microdilution method was used to determine the MIC, employing 96‐well plates and testing against Gram‐negative bacteria Escherichia coli (*E. coli*) and Gram‐positive bacteria *Bacillus subtilis* (*B. subtilis*). The cultivated bacteria in the Luria Broth (LB) medium were serially diluted to obtain an optimum concentration of 10^−8^ CFU mL^−1^. In each well of a 96‐well plate, 20 µL of bacteria, 20 µL of each synthesized sample were added having different concentrations (ranging from 6.25, 12.5, 25, 50, 100, 200, and 400 µg mL^−1^), and 160 µL of LB each. The negative consisted of inoculated broth without samples in the last wells. The plates were incubated in a microplate shaker for 24 h at 37 °C with slow shaking. For the control samples similarly, CD was tested from concentrations ranging from 6.25, 12.5, 25, 50, 100, 200, and 400 µg mL^−1^.

Four petri plates were developed using Luria Broth agar for two different microbial strains, two for *E. coli* and *B. subtilis* bacteria. For this, 100 mL of a solution with Luria Broth and agar was autoclaved, poured into the plates, and kept for solidification. After solidification, 5 mm wells were made in the plate with the help of a cork borer, and sterile spreaders were used to inoculate 50 µL bacteria on the plate with slight modifications. For decisive results, CD, CPD, and CPD‐TA and CPD‐TA: Ins with a final 200 µg mL^−1^ concentration were added to respective wells and incubated for 24 h at 37 °C. A similar protocol was followed for both strains to evaluate the zone of inhibition in the presence of light (visible light source 23 W white LED for 1 h) to determine photodynamic antibacterial annihilation activity.

### Cell Viability Studies of CD, CPD, and CPD‐TA Nanoglue

Using the MTT 3‐(4,5‐dimethylthiazol‐2‐yl)−2,5‐diphenyltetrazolium bromide) assay the effect of CPD‐TA formulation was tested for cell cytotoxicity on the HEKa cell line (Human Epidermal Keratinocytes, adult) under normal and diabetic conditions both in the presence and absence of light. In a 96‐well plate, HEKa cells were seeded (1 × 10^4^) to achieve the desired cell confluency. Samples of CD, CPD, and CPD‐TA having different concentrations (1, 5, 25, 100, 250, and 1000 µg mL^−1^) as well as insulin (1, 2, 4, 8, 16, and 32 µg mL^−1^ respectively) and CPD‐TA: Ins were added into cells in sets of three for concordant readings. The plate was incubated at 37 °C in 5% CO_2_ for 24, 48 and 72 h. The media in the MTT plate was replaced with fresh media containing MTT (2 mg mL^−1^ in 5% ethanol) and placed in the incubator for 3 h. Afterward, the media was replaced with 200 µL dimethyl sulfoxide (DMSO) to dissolve formazan crystals. A similar protocol was followed for testing the cell viability under light exposure wherein the cells were exposed to visible light (23 W white LED) for 1 h. Finally, the absorbance was measured at 575 nm, and percentage inhibition was calculated using the following equation:

(3) $$\%inhibition=\left[1-\left(A_{t}/A_{c}\right)\times 100\right]\%$$

Here, A_t_ is the absorbance of the test substance, and A_c_ is the absorbance of the control solvent for each concentration.

### Phase Contract Imaging of CPD‐TA Nanoglue for Normal and Diabetic Wound Healing Scratch Assay

To determine the potential effect of synthesized nanoformulations on wound healing in vitro, the HEKa cell line was grown in 60 mm plates with and without glucose (360 mg dL^−1^) to monitor the changes in wound healing in normal and diabetic conditions. The cells were maintained in a humidified incubator at 37 °C with 5% CO_2_ and cultured in DMEM‐F12 media without FBS. The cells were grown until they reached a confluency of 80–85%. Afterward, the wound was created in the confluent plate to analyze the healing abilities of formulations using the scratch assay. The plates were later incubated with fixed concentrations of 1 mg mL^−1^ of different samples, including CD, CPD, CPD‐TA, insulin, and CPD‐TA: Ins. Time‐lapse imaging was done to monitor the variation in wound diameter, and the changes in the wound width were measured after 0, 6, 12, and 24 h, respectively, at multiple positions of the scratch‐made in each plate. The mean of these readings at each time point was used to calculate the percentage change in wound diameter for both normal and diabetic wounds separately.

### Determination of Combination Index (CI) of CPD‐TA Nanoglue

When given together, the CI was used as one of the quantitative measures to calculate the combinatorial effect of different drugs. The drug CI was estimated when investigating synergistic or antagonistic drug combinations to quantify the level of synergism or antagonism. A CI < 1 indicated synergism, where the drugs enhanced each other's activity. Conversely, a CI > 1 suggested antagonism where one drug inhibited the action of the other and CI = 1 signified an additive effect, indicating no interference between the drugs' actions. For CI calculation the cell viability of HEKa cells was assessed at different concentrations of insulin and CPD‐TA using the following equation:

(4) $$CI=\left(D_{1}\right)/\left(D_{x1}\right)+\left(D_{2}\right)/\left(D_{x2}\right)$$

Here, *D*_1_ and *D*_2_ respectively indicate insulin and CPD‐TA concentrations. *D*_x_ was calculated using *D*_m_ [*f*_a_/*f*_u_]^1/m^. The median effect equation (*f*) determined single drug concentrations, which gave the same effect as *D*_x1_ and *D*_x2_. Equations *f*_a_ and *f*_u_ denote the cell fractions affected and unaffected, respectively. They are calculated as 10^(y‐intercept)/^ *^m^*, where *m* represents the slope median in the median effect plot of log (*D*) versus log (*f*_a_/*f*_u_).

### Statistical Analysis

The data is the mean ± SD of at least three independent experiments. The statistical data analysis was done in MS Excel using one‐way ANOVA. The corresponding *p*‐values were calculated to check whether the data was statistically significant.

## Conflict of Interest

The authors declare no conflict of interest.

## Author Contributions

M.A. performed conceptualization, methodology, experimentation, validation, analysis, data interpretation, and visualization and wrote the original draft. D.S. performed biological analysis, data interpretation, and visualization and wrote the original draft. S.S. performed experimentation, analysis, and data interpretation. D.K.K. performed thermal and mechanical studies, and supervision, acquired resources, and wrote, reviewed, and edited the final draft D.C. performed conceptualization, resources, visualization, and supervision, and wrote, reviewed, and edited the final draft. P.D. performed conceptualization, visualization, and supervision, acquired resources, and wrote reviewed, and edited the final draft.

## Supporting information

Supporting Information

## Data Availability

The data that support the findings of this study are available in the supplementary material of this article.

## References

[1] H. Wang , X. Ke , S. Tang , K. Ren , Q. Chen , C. Li , W. Ran , C. Ding , J. Yang , J. Luo , J. Li , Small 2024, 20, 2307628. 10.1002/smll.202307628 38191883

[2] D. G. Barrett , G. G. Bushnell , P. B. Messersmith , Adv. Healthc. Mater. 2013, 2, 745. 23184616 10.1002/adhm.201200316 PMC3685437

[3] J. Li , A. D. Celiz , J. Yang , Q. Yang , I. Wamala , W. Whyte , B. R. Seo , N. V. Vasilyev , J. J. Vlassak , Z. Suo , D. J. Mooney , Science 2017, 357, 378. 28751604 10.1126/science.aah6362 PMC5905340

[4] A. D. Roberts , W. Finnigan , P. P. Kelly , M. Faulkner , R. Breitling , E. Takano , N. S. Scrutton , J. J. Blaker , S. Hay , Mater. Today Bio 2020, 7, 100068. 10.1016/j.mtbio.2020.100068 PMC7366031 32695986

[5] Y. Qian , K. Xu , L. Shen , M. Dai , Z. Zhao , Y. Zheng , H. Wang , H. Xie , X. Wu , D. Xiao , Q. Zheng , J. Zhang , Y. Song , J. Shen , W. Chen , Adv. Funct. Mater. 2023, 33, 2300707.

[6] D. A. Belcher , A. T. Williams , A. F. Palmer , P. Cabrales , Sci. Rep. 2021, 11, 10834. 34035380 10.1038/s41598-021-90431-z PMC8149844

[7] Y. Huang , W. Jing , J. Zeng , Y. Xue , Y. Zhang , X. Yu , P. Wei , B. Zhao , J. Dong , Adv. Healthc. Mater. 2023, 12, 2301086. 10.1002/adhm.202301086 37421335

[8] H.‐W. Sung , D.‐M. Huang , W.‐H. Chang , R.‐N. Huang , J.‐C. Hsu , J. Biomed. Mater. Res. 1999, 46, 520. 10398013 10.1002/(sici)1097-4636(19990915)46:4<520::aid-jbm10>3.0.co;2-9

[9] L. Fan , X. Zhang , L. Wang , Y. Song , K. Yi , X. Wang , H. Zhang , L. Li , Y. Zhao , Adv. Funct. Mater. 2024, 34, 2316742

[10] H. Li , Y. Shi , W. Zhang , M. Yu , X. Chen , M. Kong , ACS Appl. Mater. Interfaces 2022, 14, 18097. 35417132 10.1021/acsami.2c00236

[11] H. Montazerian , R. R. Sampath , N. Annabi , A. Khademhosseini , P. S. Weiss , Acc. Mater. Res. 2023, 4, 627.

[12] D. Zhou , S. Li , M. Pei , H. Yang , S. Gu , Y. Tao , D. Ye , Y. Zhou , W. Xu , P. Xiao , ACS Appl. Mater. Interfaces 2020, 12, 18225. 32227982 10.1021/acsami.9b22120

[13] M. Aggarwal , H. Panigrahi , D. K. Kotnees , P. Das , Biomacromolecules 2024, 25, 3178. 38632677 10.1021/acs.biomac.4c00313

[14] S. Tao , T. Feng , C. Zheng , S. Zhu , B. Yang , J. Phys. Chem. Lett. 2019, 10, 5182. 31424936 10.1021/acs.jpclett.9b01384

[15] C. Xia , S. Tao , S. Zhu , Y. Song , T. Feng , Q. Zeng , J. Liu , B. Yang , Chem. Eur. J. 2018, 24, 11303. 29904946 10.1002/chem.201802712

[16] S. Mandal , H. Panigrahi , K. Dinesh Kumar , P. Das , Polymer 2022, 254, 125102.

[17] R. Pinnaratip , M. S. A. Bhuiyan , K. Meyers , R. M. Rajachar , B. P. Lee , Adv. Healthc. Mater. 2019, 8, 1801568. 10.1002/adhm.201801568 PMC6636851 30945459

[18] T. Xu , X. Zhang , H. Yang , H. Li , S. Zhang , Z. Yang , X. Jia , X. Liu , J. Li , Chem. Eng. J. 2023, 467, 143465.

[19] T. Feng , S. Zhu , Q. Zeng , S. Lu , S. Tao , J. Liu , B. Yang , ACS Appl. Mater. Interfaces 2018, 10, 12262. 29164859 10.1021/acsami.7b14857

[20] Y.‐T. Gao , B.‐B. Chen , L. Jiang , J. Lv , S. Chang , Y. Wang , R.‐C. Qian , D.‐W. Li , M. E. Hafez , ACS Appl. Mater. Interfaces 2021, 13, 50228. 34651499 10.1021/acsami.1c12993

[21] S. A. Hill , D. Benito‐Alifonso , D. J. Morgan , S. A. Davis , M. Berry , M. C. Galan , Nanoscale 2016, 8, 18630. 27801469 10.1039/c6nr07336k

[22] R. Yasmin , M. Shah , S. A. Khan , R. Ali , Nanotechnol. Rev. 2017, 6, 191.

[23] J. Guo , W. Sun , J. P. Kim , X. Lu , Q. Li , M. Lin , O. Mrowczynski , E. B. Rizk , J. Cheng , G. Qian , J. Yang , Acta Biomater. 2018, 72, 35. 29555464 10.1016/j.actbio.2018.03.008 PMC6328059

[24] Z. Hao , G. Liu , L. Ren , J. Liu , C. Liu , T. Yang , X. Wu , X. Zhang , L. Yang , J. Xia , W. Li , ACS Appl. Mater. Interfaces 2023, 15, 19847. 37042619 10.1021/acsami.2c23323

[25] D. Sharda , D. Choudhury , RSC Adv. 2023, 13, 20321. 37425626 10.1039/d3ra01473h PMC10323873

[26] B. Wu , W. Pan , S. Luo , X. Luo , Y. Zhao , Q. Xiu , M. Zhong , Z. Wang , T. Liao , N. Li , C. Liu , C. Nie , G. Yi , S. Lin , M. Zou , B. Li , L. Zheng , Adv. Sci. 2024, 11, 2306711. 10.1002/advs.202307630 PMC11095230 38441389

[27] Z. Guo , Z. Zhang , N. Zhang , W. Gao , J. Li , Y. Pu , B. He , J. Xie , Bioact. Mater. 2022, 15, 203. 35386343 10.1016/j.bioactmat.2021.11.036 PMC8940763

[28] D. Sharda , D. Choudhury , Mater. Adv. 2024, 5, 5231.

[29] Y. Wu , Y. Lyu , L. Li , K. Zhou , J. Cai , X. Wang , H. Wang , F. Yan , Z. Weng , Biomacromolecules 2024, 25, 43. 38141019 10.1021/acs.biomac.3c00698

[30] H. Guo , S. Huang , A. Xu , W. Xue , Chem. Mater. 2022, 34, 2655.

[31] Y. Guo , S. Ding , C. Shang , C. Zhang , M. Li , Q. Zhang , L. Gu , B. C. Heng , S. Zhang , F. Mei , Y. Huang , X. Zhang , M. Xu , J. Jiang , S. Guo , X. Deng , L. Chen , Adv. Mater. 2023, 36, 2306292. 10.1002/adma.202306292 37723937

[32] C. Fu , Y. Fan , G. Liu , W. Li , J. Ma , J. Xiao , Chem. Eng. J. 2024, 480, 148288.

[33] X. Huang , L. Zheng , Y. Zhou , S. Hu , W. Ning , S. Li , Z. Lin , S. Huang , Adv. Healthc. Mater. 2024, 13, 2302256. 10.1002/adhm.202302256 37922497

[34] Q. Li , X. Shen , D. Xing , Dye Pigm. 2023, 208, 110784.

[35] S. Zhang , Z. Yang , J. Hao , F. Ding , Z. Li , X. Ren , Chem. Eng. J. 2022, 432, 134309.

[36] H. Chen , Y. Guo , Z. Zhang , W. Mao , C. Shen , W. Xiong , Y. Yao , X. Zhao , Y. Hu , Z. Zou , J. Wu , Nano Lett. 2022, 22, 229. 34928162 10.1021/acs.nanolett.1c03693

[37] X. Qian , T. Lu , C. Huang , D. Zheng , G. Gong , X. Chu , X. Wang , H. Lai , L. Ma , L. Jiang , X. Sun , X. Ji , M. Li , Y. Zhang , Adv. Funct. Mater. 2024, 34, 2315576.

[38] Y. Yan , L. Wei , J. Shao , X. Qiu , X. Zhang , X. Cui , J. Huang , S. Ge , Small 2024, 20, 2310870. 10.1002/smll.202310870 38453669

[39] H. Liu , J. Wang , Y. Deng , G. Zou , J. Xu , Endocr. J. 2021, 68, EJ20. 10.1507/endocrj.EJ20-0575 33867397

[40] D. Sharda , S. Ghosh , P. Kaur , B. Basu , D. Choudhury , Discov. Nano 2023, 18, 154. 38087141 10.1186/s11671-023-03941-2 PMC10716098

[41] M. Tan , J. Zeng , F.‐Z. Zhang , Y.‐T. Zhang , H. Li , S.‐T. Fan , J.‐X. Wang , M. Yuan , B.‐J. Li , S. Zhang , ACS Appl. Mater. Interfaces 2023, 15, 50809. 10.1021/acsami.3c10607 37889121

[42] C. Dunnill , T. Patton , J. Brennan , J. Barrett , M. Dryden , J. Cooke , D. Leaper , N. T. Georgopoulos , Int. Wound J. 2017, 14, 89. 26688157 10.1111/iwj.12557 PMC7950185

[43] X. Liu , W. Dan , H. Ju , N. Dan , J. Gong , RSC Adv. 2015, 5, 52079.

[44] S. Kumari , A. Solanki , S. Mandal , D. Subramanyam , P. Das , Bioconjug. Chem. 2018, 29, 1500. 29634254 10.1021/acs.bioconjchem.8b00173 PMC7116061

[45] B. Cai , L. Rao , X. Ji , L. Bu , Z. He , D. Wan , Y. Yang , W. Liu , S. Guo , X. Zhao , J. Biomed. Mater. Res. Part A 2016, 104, 2854. 10.1002/jbm.a.35823 27376586

[46] S. K. Bhattacharyya , M. Dule , R. Paul , J. Dash , M. Anas , T. K. Mandal , P. Das , N. C. Das , S. Banerjee , ACS Biomater. Sci. Eng. 2020, 6, 5662. 33320568 10.1021/acsbiomaterials.0c00982

[47] B. Yang , S. Jin , Y. Park , Y. M. Jung , H. J. Cha , Small 2018, 14, 1803377. 10.1002/smll.201803377 30457699

[48] R. Bitton , E. Josef , I. Shimshelashvili , K. Shapira , D. Seliktar , H. Bianco‐Peled , Acta Biomater. 2009, 5, 1582. 19272847 10.1016/j.actbio.2008.10.004

[49] R. M. LoPachin , T. Gavin , Chem. Res. Toxicol. 2014, 27, 1081. 24911545 10.1021/tx5001046 PMC4106693

[50] Z. Tong , W. He , X. Fan , A. Guo , Front. Vet. Sci. 2022, 8, 803657. 35083309 10.3389/fvets.2021.803657 PMC8784788

