# Supplementary material for: Carbonized Polymer Dot‐Tannic Acid Nanoglue: Tissue Reinforcement with Concurrent Fluorescent Tracking, Insulin Delivery, and Reactive Oxygen Species Regulation for Normal and Diabetic Wound Healing
Source: Small. 2024 Aug 15;20(47):2405531. doi: 10.1002/smll.202405531 (PMC11579962; doi:10.1002/smll.202405531)
Supplement: Supplementary file 1 — Supporting Information [file SMLL-20-2405531-s001.docx]

**Supporting Information**

**Carbonized Polymer Dot-Tannic Acid Nanoglue: Tissue reinforcement with Concurrent Fluorescent Tracking, Insulin Delivery, and Reactive Oxygen Species Regulation for Normal and Diabetic Wound Healing**

Maansi Aggarwal^a^, Deepinder Sharda^b^, Shruti Srivastava^a^, Dinesh Kumar Kotnees^c^, Diptiman Choudhury^bd*^, Prolay Das^a*^

^a^Department of Chemistry, Indian Institute of Technology Patna, Patna 801103, Bihar, India

^b^Department of Chemistry and Biochemistry, Thapar Institute of Engineering and Technology (TIET), Patiala 147004, Punjab, India

^c^Department of Metallurgical and Materials Engineering, Indian Institute of Technology Patna, Patna 801103, Bihar, India
^d^Center of Excellence in Emerging Materials (CEEMS), Thapar Institute of Engineering and Technology, Patiala, Punjab 147004, India

^*^ Corresponding Author: [prolay@iitp.ac.in](mailto:prolay@iitp.ac.in), diptiman@thapar.edu

**Reagents and Materials**

Tannic acid, Ammonium persulphate, and Phloroglucinol were procured from Sisco Research Laboratories Pvt. Ltd., India, and used without purification. DL Dopa, Methanol, parabenzoquinone, Na_2_-EDTA and Glyoxal are purchased from SpectroChem, India, and gelatin from Himedia, India. ABTS, Dihydrorhodamine123, and Benzoylated dialysis membrane with cutoff MWCO 2 kDa and D_2_O purchased from Sigma-Aldrich, India. Normal, Human, Adult (HEKa) Cells were procured from ATCC PCS-200-011. All the experiments were performed with Milli-Q water unless specified.

**Morphological, Physicochemical, and Photophysical Characterization of CD, CPD, CPD-TA**

Purified CDs, subsequent CPD, and CPD-TA powdered X-ray diffraction (XRD) were performed on a Rigaku TTRX-III diffractometer with Cu K_α_ source (λ = 1.54 Å). For transmission electron microscopy (TEM) imaging, all three samples were drop-cast onto carbon-coated copper TEM grids and dried at room temperature under vacuum. High-resolution TEM (HRTEM) images and Selected Area Electron Diffraction (SAED) patterns were obtained using a JEM-F200 multipurpose electron microscope (Jeol) operating at 100 kV. Lyophilized samples were coated with Au for field emission scanning electron microscopy (FESEM) imaging, performed using a Gemini SEM500 microscope (ZEISS). Furthermore, the hydrodynamic radius of CD, CPD, and CPD-TA nanoglue was determined using dynamic light scattering (DLS) with water diluent on Beckman Coulter’s Delsa instrument. Zeta potential electrophoretic light scattering data were obtained from the Litesizer 500 analyzer (Anton Paar).

The surface functional groups of the samples were analyzed using Fourier Transform Infrared Spectroscopy (FTIR) on a Perkin Elmar Spectrum 400 instrument. Spectra were obtained from KBr pellets scanned in the 4000 to 400 cm^-1^ range. Additionally, the presence of -CHO groups on the CD was confirmed through nuclear magnetic resonance (NMR) analysis, including ^1^H NMR and ^13^C NMR, conducted on a Jeol 500 MHz spectrometer in D_2_O- d_2_ solvent. To confirm imine formation, Solid-state 1H NMR and 13C NMR analyses were performed on CPD and CPD-TA nanoglue. Quantifying the -CHO groups on CD was accomplished using a Schiff base reagent by measuring the intensity of the resulting pink color.

UV-visible absorption spectra were recorded using a UV-2550 spectrophotometer (Shimadzu, Japan). In contrast, steady-state fluorescence spectra for CD and conjugated bioadhesive were acquired with a Horiba Jobin Yvon Fluoromax-4P spectrofluorometer. Time-resolved fluorescence spectra were obtained using time-correlated single photon counting (TCSPC) on a picosecond scale, employing a LifeSpec-II time-resolved fluorescence spectrophotometer (Edinburgh Instruments, UK) with a 360 nm laser. Far UV data was collected using a JASCO 150 circular dichroism spectrometer, covering the 185-300 nm range at a scan rate of 100 nm/min. Measurements were conducted in a cuvette with a path length of 1 cm.

**Thermal and Mechanical Studies CPD and CPD-TA**

Thermal gravimetric analysis (TGA) was conducted using a TA Instruments Q600 SDT at a heating rate of 10 °C/min under an N_2_ atmosphere, with temperatures ranging from 25 to 1000 °C. Rheological studies and probe tack tests were performed using a Modular Compact Rheometer (MCR302, Anton Paar, Austria) at ambient temperature (25 °C). Two parallel steel plates with 8 mm diameter each and 1 mm spacing were utilized for the tack test, and adhesive samples were mounted on a mobile arm probe. Force-displacement measurements were recorded during bonding and debonding at a preselected rate of 0.1 mm/s, with force-displacement sensor measurements taken at 10 secs intervals and a constant separation rate of v = -0.1 mm/s. The rheological behavior of the CPD-TA nanoglue variants was thoroughly investigated to understand their viscoelastic properties. This analysis was performed using a Modular Compact Rheometer (MCR302, Anton Paar, Austria) configured in a parallel plate setup, ensuring precise control over the test conditions. Frequency sweep experiments were performed across a range of oscillation frequencies (0.1 –100 Hz) at a constant shear strain of 0.01% chosen from the linear viscoelastic region of the strain sweep curves. The experiments were conducted at a fixed temperature of 25°C. Such a methodology was essential for evaluating the samples' responses to varying dynamic stresses, revealing their structural and functional integrity under conditions mimicking real-world applications. Lap shear studies with CPD and CPD-TA on porcine skin models were conducted using a Zwick-Roell Z010 universal testing machine (Germany). The treated porcine skin was cut into rectangular halves (30 mm x 15 mm) and coated with nanoglue, with a similar piece of porcine skin applied with constant pressure for 30 mins.

**Agarose Gel Electrophoresis**

Nanoglue transformation was determined in a Bio-Rad Electrophoresis system with 1% Agarose gel in 1X Tris-Acetate-EDTA (TAE) buffer. Following the standard protocol, the gel was electrophoresed at 90 V for 1.5 h. Gel imaging was performed using a Canon digital camera on a UV illuminator.

**Ninhydrin Assay**

The presence of primary amine groups on the surface of CD was assessed using a Ninhydrin colorimetric assay. Standard amino acid solutions and CD samples were prepared, and 100 µL of 8% Ninhydrin reagent was added. After thorough mixing, the tubes were incubated in a water bath for 10 min. The reaction was stopped by adding 300 µL of ethanol and absorbance was measured at 570 nm. -NH_2_ concentrations on the CD were determined using a standard curve.

**Schiff Base Assay**

We developed a Schiff base assay to quantify -CHO groups on the CD surface. The assay relies on a calorimetric determination method, where the intensity of the pink color indicates the presence of aldehyde groups. For the reaction standard, 40% glyoxal solutions were prepared at different concentration ranges (0.5- 500 mM). CD (10 µL), CPD (10 mg) and CPD-TA (10 mg) samples were dissolved in 1 mL of distilled water. To each mixture, Schiff assay (10% v/v) was added (200 µL) with 10 µL of all the samples to form aliquots of 400 µL and incubated for 20 mins to achieve uniform coloration. The concentration of aldehyde groups on CD was determined by constructing a standard UV-visible curve and measuring the absorbance at 550 nm.

**Synthesis of CPD with Gelatin and Organic Precursor**

A comparative study assessed the efficiency and properties of CPDs synthesized through a two-step hydrothermal process versus the traditional single-step hydrothermal process. In the latter, a mixture containing 1/4^th^ of the CD precursors (glyoxal: 0.75 mmol, phloroglucinol: 0.125 mmol, and DL-Dopa: 0.25 mmol) and the same concentration of gelatin (30% w/v in 4 mL water) subjected to hydrothermal treatment at 150 °C for 8 h within a Teflon-lined autoclave. Subsequently, the volume of the resulting CPD was reduced to 2 mL, and its photophysical characteristics and rheological were evaluated.

**Porcine Skin Preparation for Tensile Strength Test**

Fresh porcine skin was obtained from a local slaughterhouse for the lap shear test. The dorsal sections were cut into two pieces measuring 30 mm × 15 mm and were thoroughly washed with warm water. Subsequently, the subcutaneous fat layer was removed using a surgical scalpel, and the dermal layer was meticulously cleaned with gauze and isopropyl alcohol following the standard protocol (ASTM F2255-2015). The tissue was soaked in a 0.9% saline solution to maintain moisture retention and preservation. The prepared nanoglue was evenly applied onto the tissue with an overlapping region of 15 mm × 15 mm and allowed to dry at room temperature for 30 mins.

**
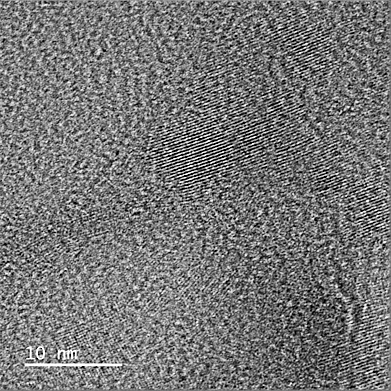
**

**Figure S1.** HRTEM image of CD with lattice spacing to be 0.22 nm.





**Figure S2.** pXRD spectra of CD showcasing semi-amorphous nature.


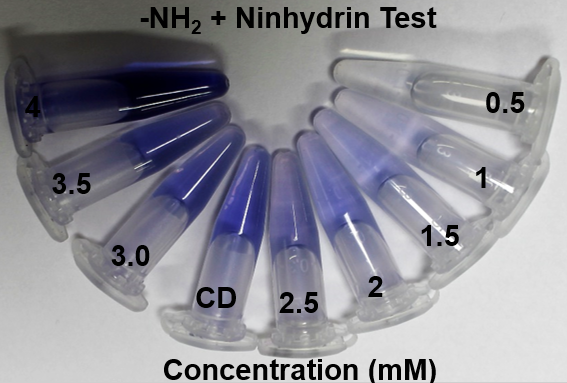


**Figure S3.** Digital images of the Ninhydrin test at different amino acid concentrations for determining -NH_2_ content in CD.


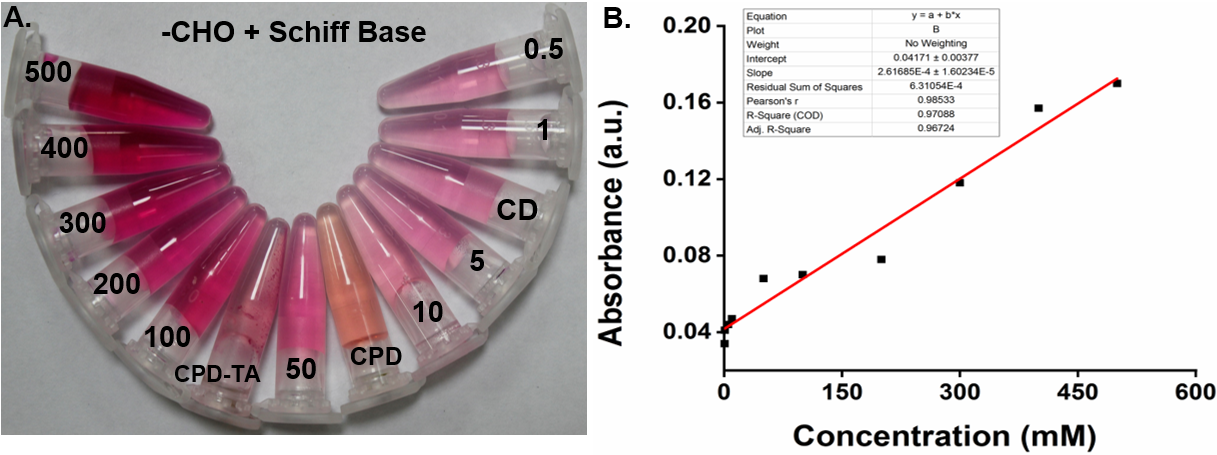


**Figure S4.** A. Digital images of Schiff base assay at different glyoxal concentrations and B. Jobs plot determining -CHO concentration to 4.02 mM in 10 µL CD.


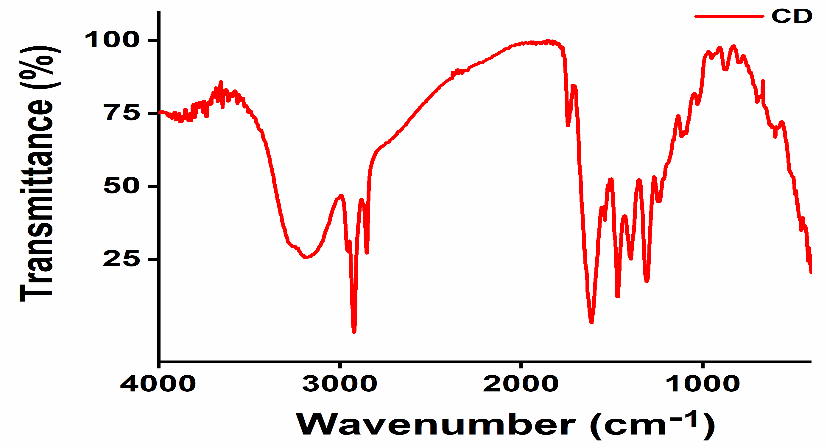


**Figure S5.** Aldehyde-rich CD functionalities using FTIR spectra.

**
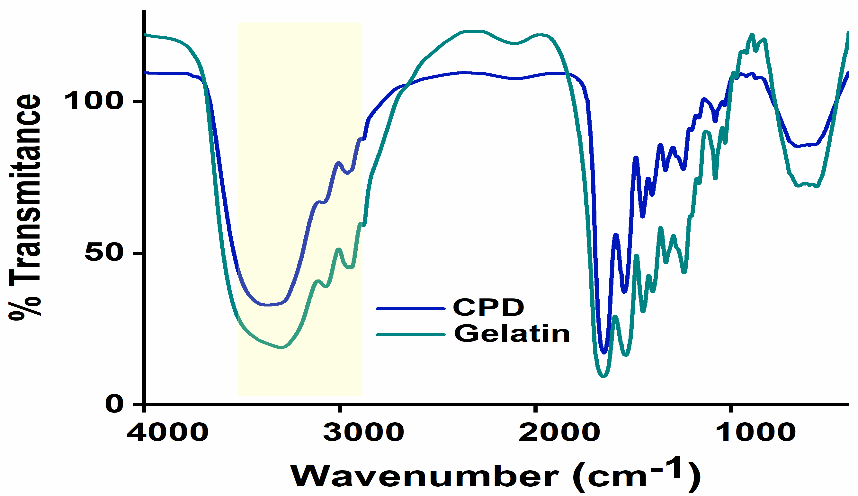
**

**Figure S6.** FTIR spectra confirm that CPD is upholding integral functionalities of gelatin.


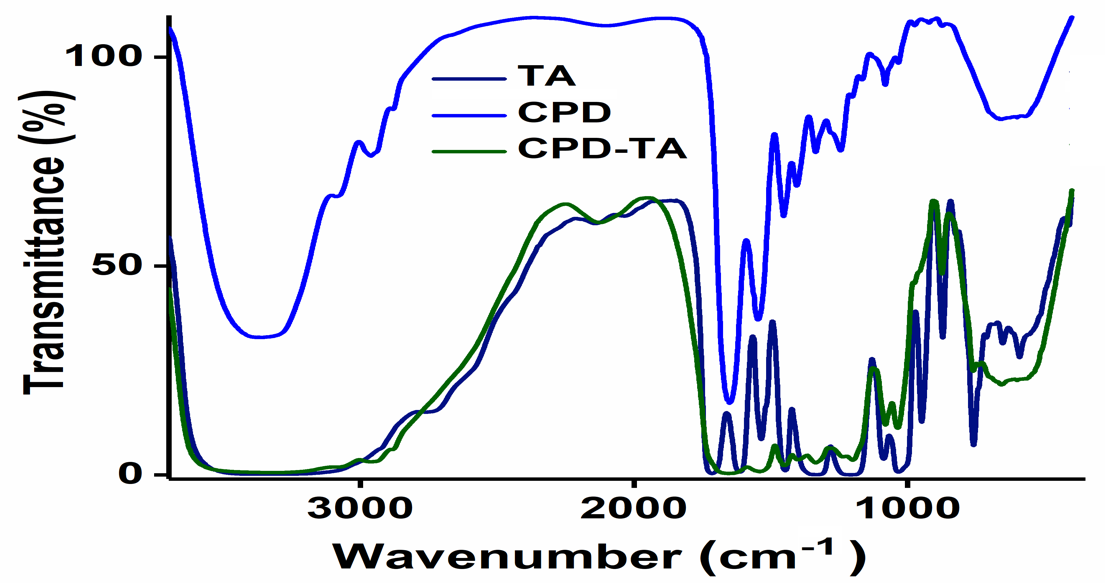


**Figure S7.** FTIR spectra showcasing successful grafting of TA on CPD at 1036 cm^–1^.

**
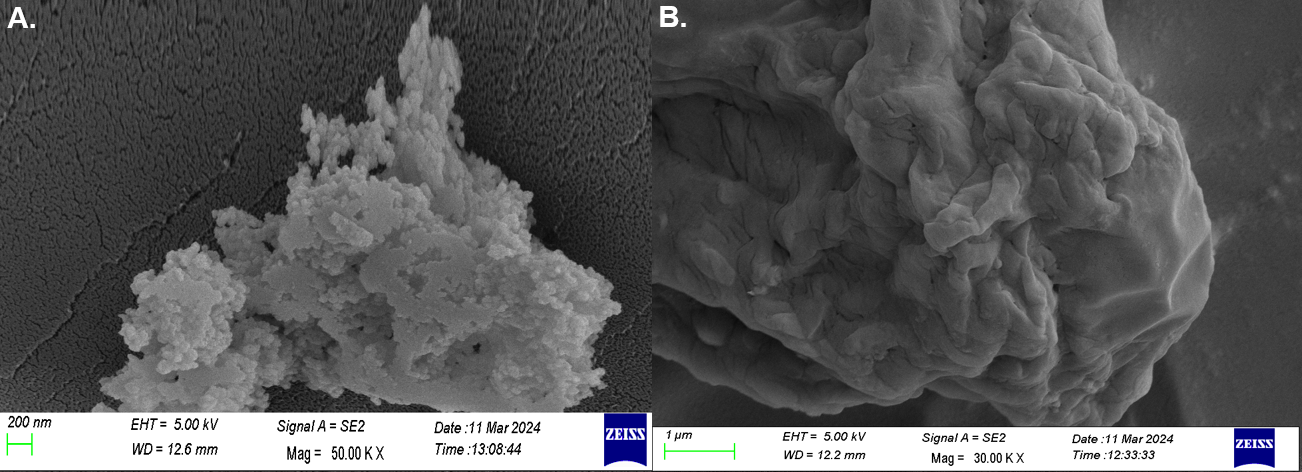
**

**Figure S8.** FESEM image A. CPD-TA (0.15 g) with an average diameter of 100 nm and B. CPD-TA (0.40 g) with highly diffused structure.

**
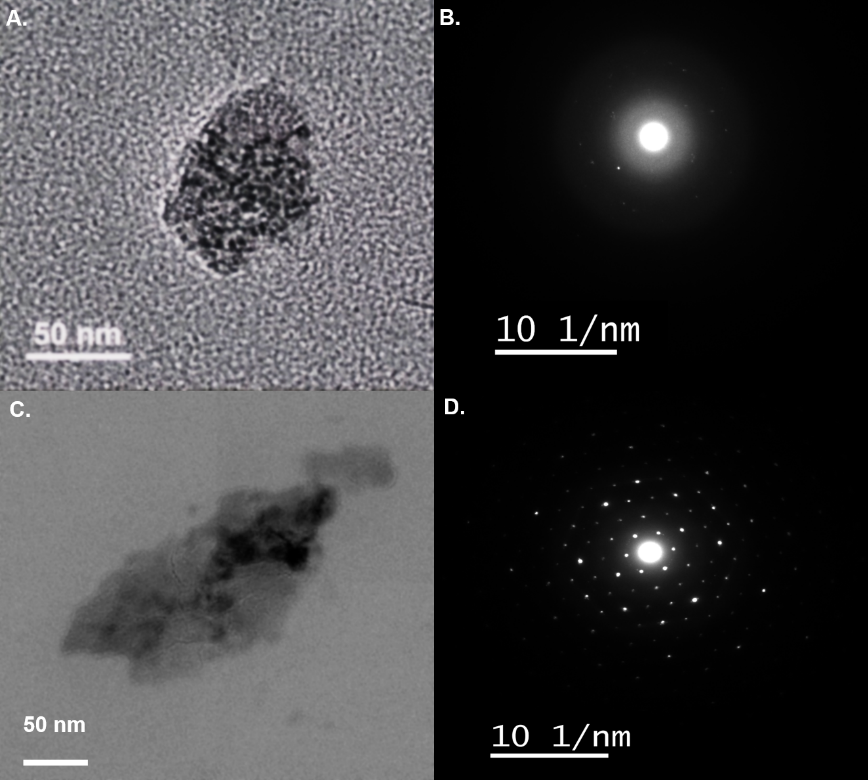
**

**Figure S9.** A-B. CPD TEM image and corresponding SAED pattern depicting semi-amorphous nature and C-D. The TEM image and corresponding SAED pattern depict the crystalline nature of CPD.

**
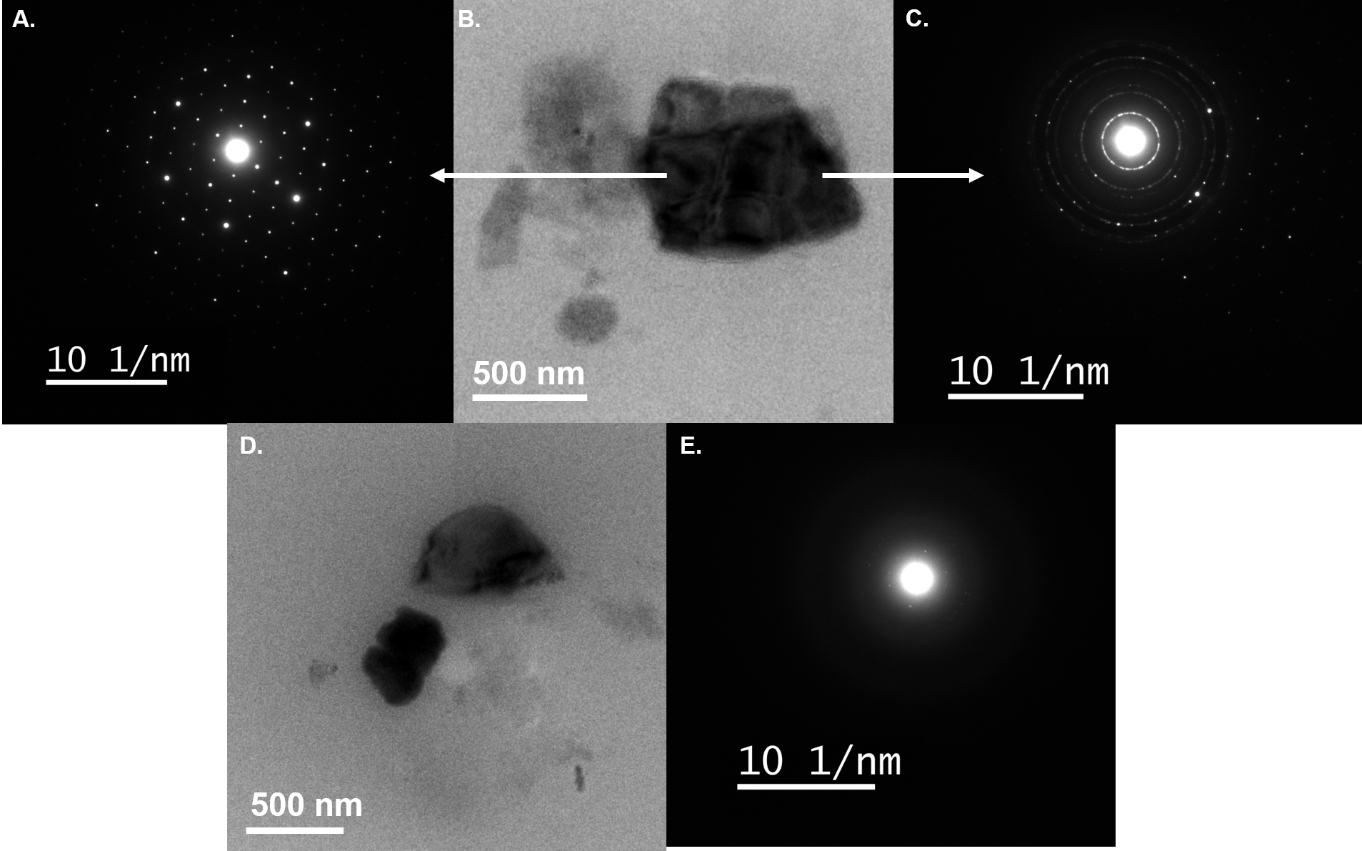
**

**Figure S10.** A-C. CPD-TA TEM image and corresponding SAED patterns indicate both semi-amorphous and crystalline nature and D-E. TEM image and corresponding SAED patterns depict the amorphous nature of CPD-TA.

**
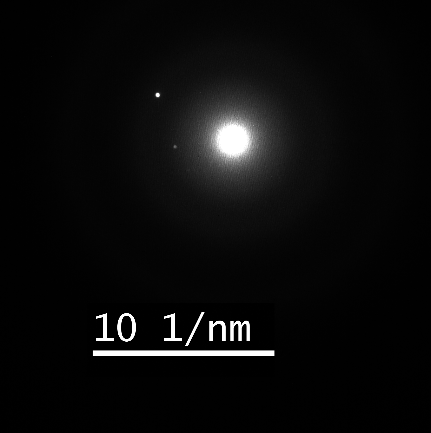
**

**Figure S11.** A.CD SAED patterns indicate a semi-amorphous nature.

**
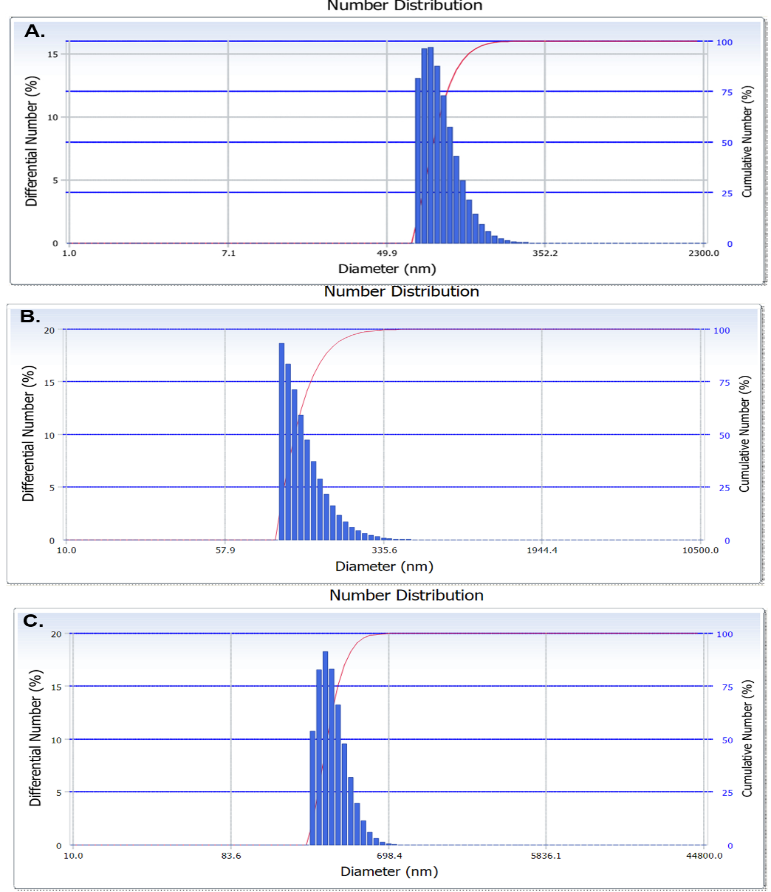
**

**Figure S12.** DLS spectra with an average diameter of A. CD: 52 nm, B. CPD: 89 nm and C. CPD-TA: 343 nm.

**
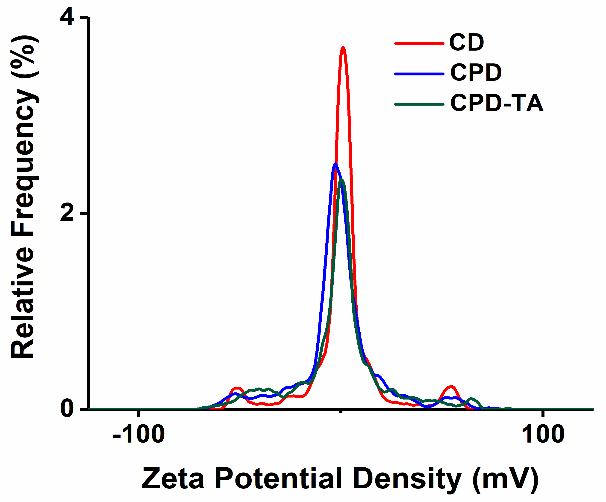
**

**Figure S13.** Mean zeta potential of CD (1.13 mV), CPD (-3.3 mV), and CPD-TA (-1.4 mV).


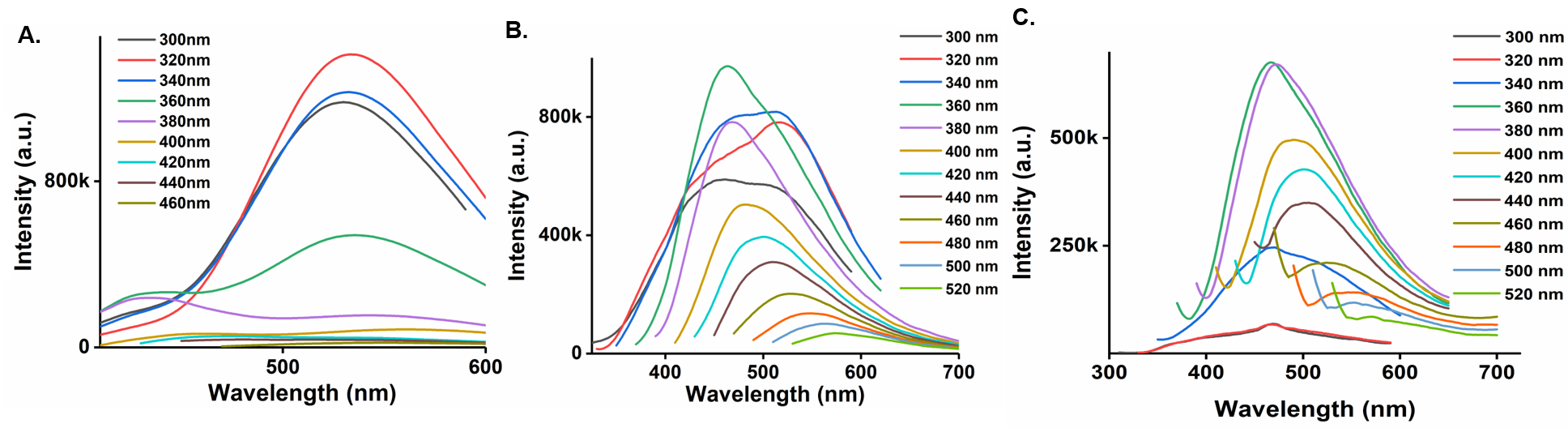


**Figure S14.** Photoluminescence spectra A. CD, B. CPD and C. CPD-TA.

**Table S1.** Biexpontial values of lifetime for CD, CPD and CPD-TA.

|  | **Lifetime (ns)** | | **Pre exponential factor** | |  |
| --- | --- | --- | --- | --- | --- |
|  | **Ꚍ_1_** | **Ꚍ_2_** | **B_1_** | **B_2_** | **χ^2^** |
| **CD** | 1.6924 | 8.6510 | 0.082 | 0.030 | 1.119 |
| **CPD** | 1.0609 | 5.1473 | 0.116 | 0.010 | 1.052 |
| **CPD-TA** | 0.7993 | 5.1197 | 0.107 | 0.015 | 1.287 |

**
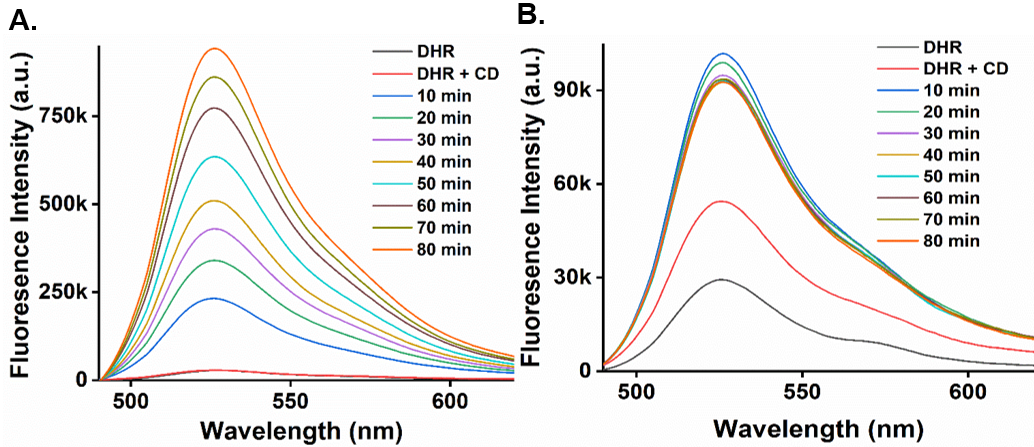
**

**Figure S15.** A. Fluorescence spectra at 480 nm excitation for CD in the presence of DHR123 converting to Rhodamine 123 under visible light confirming ROS generation and B. Activity under dark conditions (without light).

**
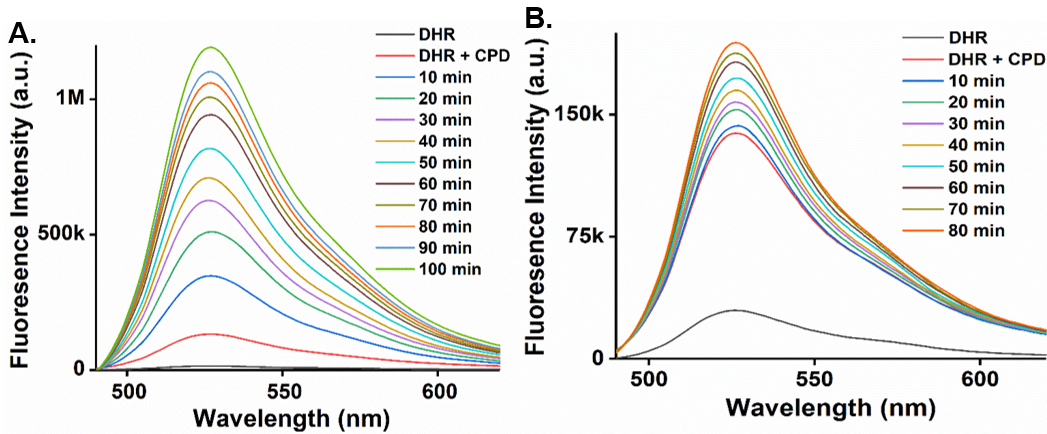
**

**Figure S16.** A. Fluorescence spectra at 480 nm excitation for CPD in the presence of DHR123 converting to Rhodamine 123 under visible light confirming ROS generation and B. Activity under dark conditions (without light).

**
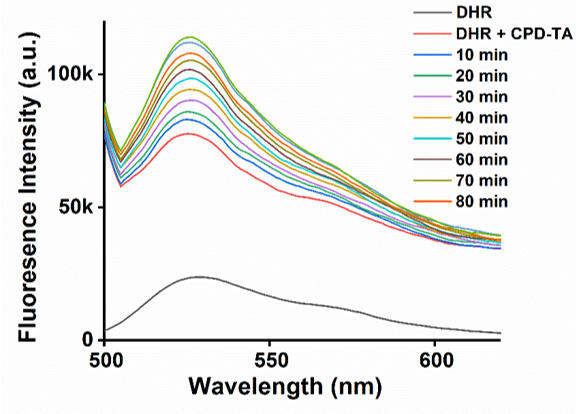
**

**Figure S17.** Fluorescence spectra at 480 nm excitation of DHR123 activity of CPD-TA under dark conditions (without light).

**
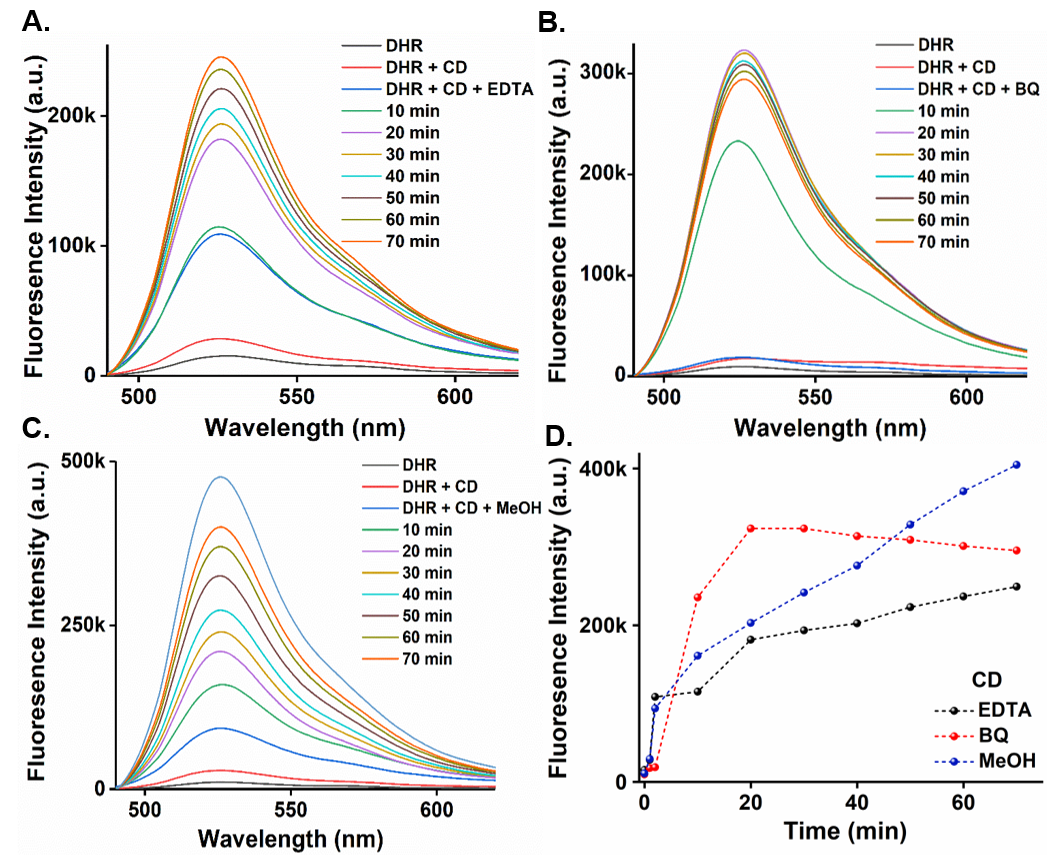
**

**Figure S18.** CD A. Trapping of surface generated h^+^ using Na_2_-EDTA under visible light, B. Trapping of O^2−^ ﻿using BQ under visible light, C. Trapping of ^.^OH ﻿using MeOH under visible light and D. Combine changes in free radical trapping suggesting surface generated h^+^ as major ROS species.

**
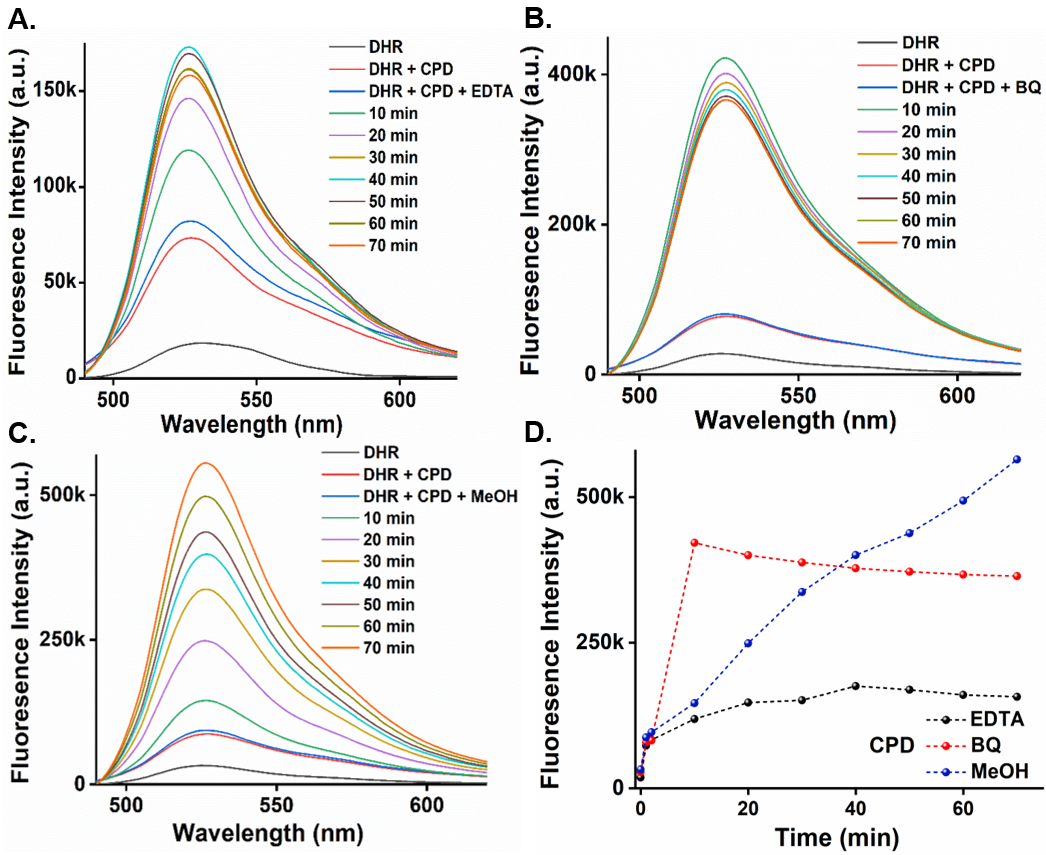
**

**Figure S19.** CPD A. Trapping of surface generated h^+^ using Na_2_-EDTA under visible light, B. Trapping of O^2−^ ﻿using BQ under visible light, C. Trapping of ^.^OH ﻿using MeOH under visible light and D. Comparison w.r.t fluorescence intensity for free radical trapping suggesting surface generated h^+^ as major ROS species.


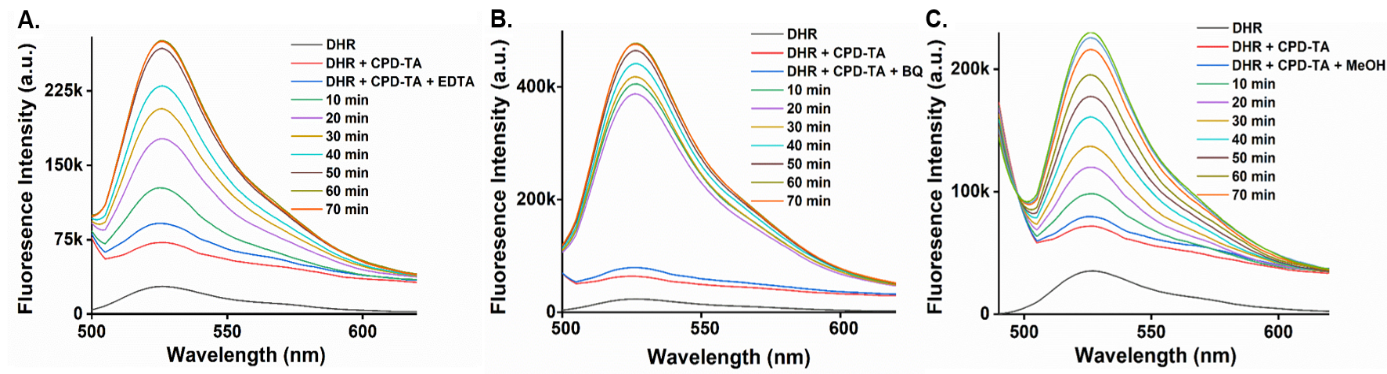


**Figure S20.** CPD-TA A. Trapping of surface generated h^+^ using Na_2_-EDTA under visible light, B. Trapping of O^2−^ ﻿using BQ under visible light, and C. Trapping of ^.^OH ﻿using MeOH under visible light.


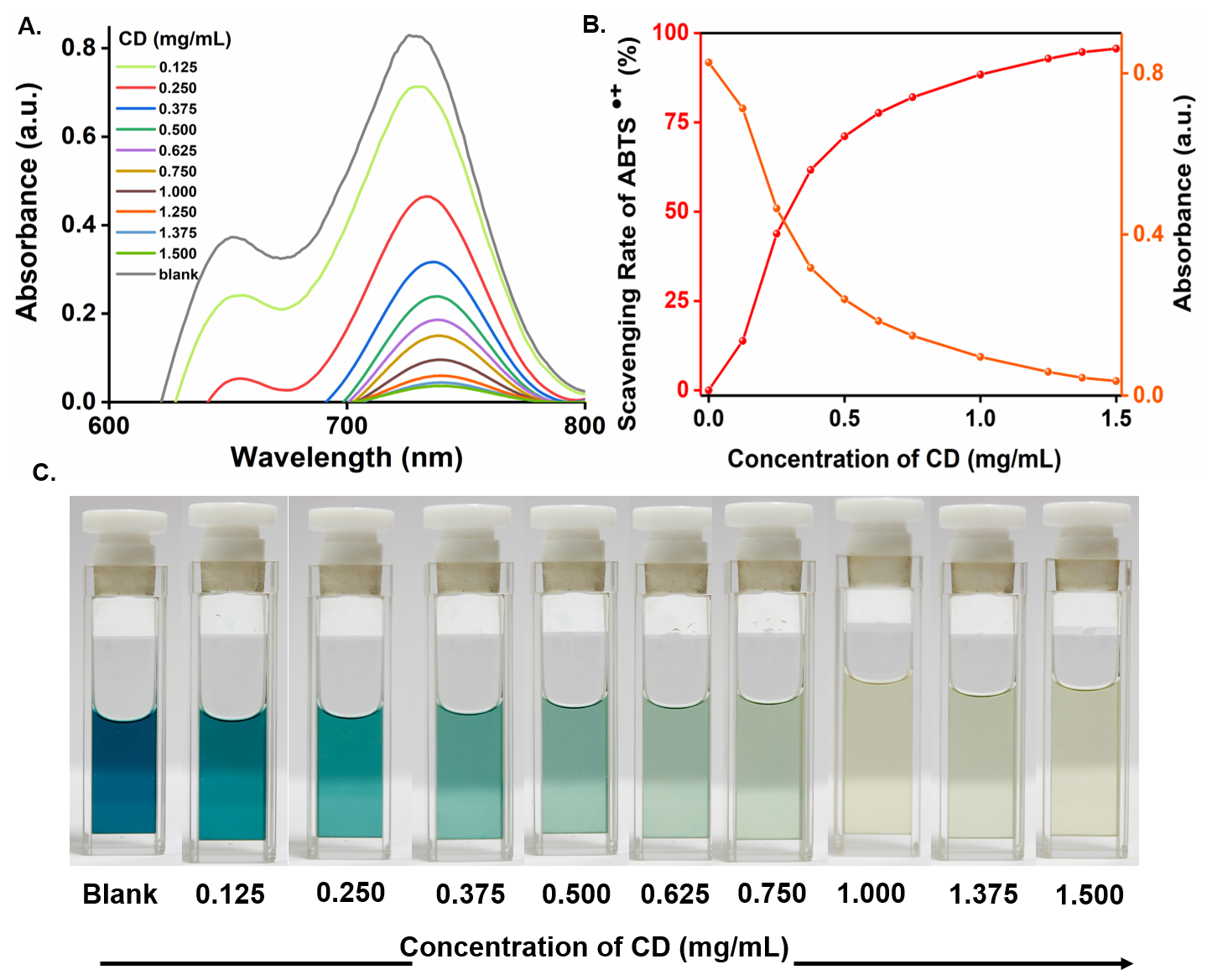


**Figure S21.** A. UV-Visible spectra of ABTS assay with varying concentration of CD (mg/mL), B. Scavenging rate of ABTS**^˙+^** vs concentration of CD relation with absorbance and C. Digital images of decolourising ABTS**^˙+^** assay.


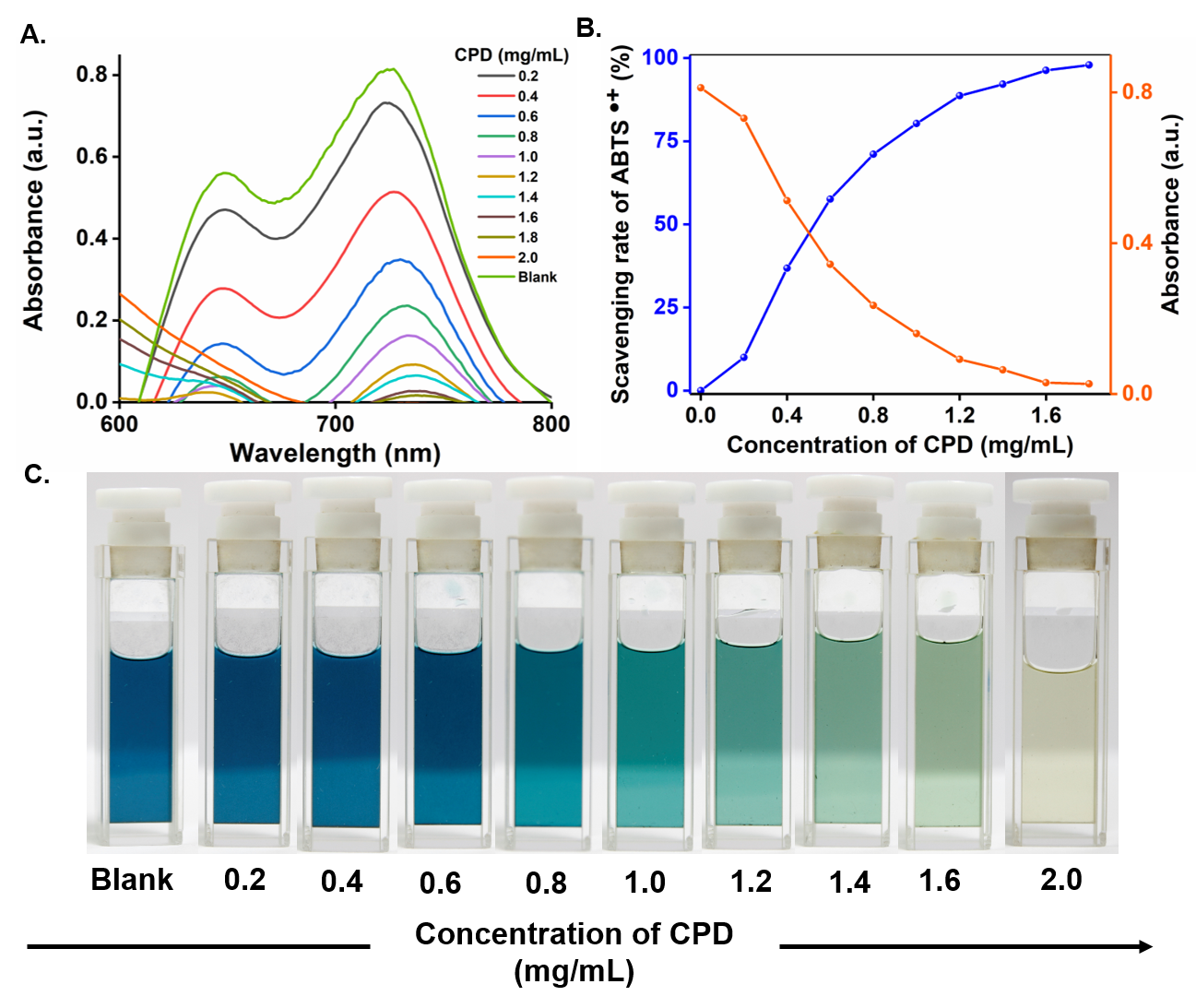


**Figure S22.** A. UV-Visible spectra of ABTS assay with varying concentration of CPD (mg/mL), B. Scavenging rate of ABTS**^˙+^** vs concentration of CPD relation with absorbance and C. Digital images of decolourising ABTS**^˙+^** assay.

**
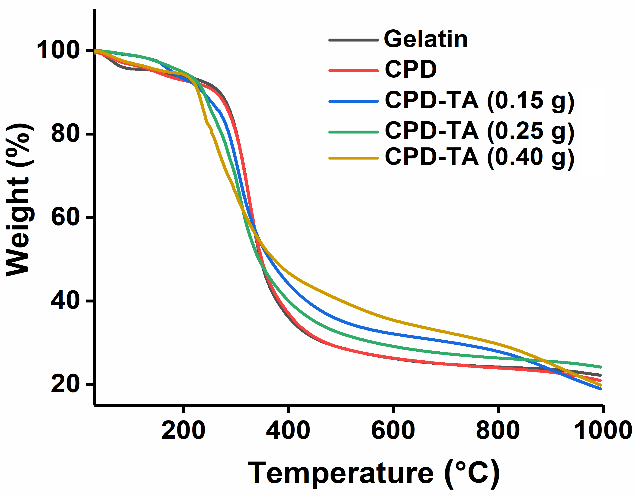
**

**Figure S23.** TGA plot with enhanced thermal resilience imparted by incorporating TA into the CPD matrix.

**
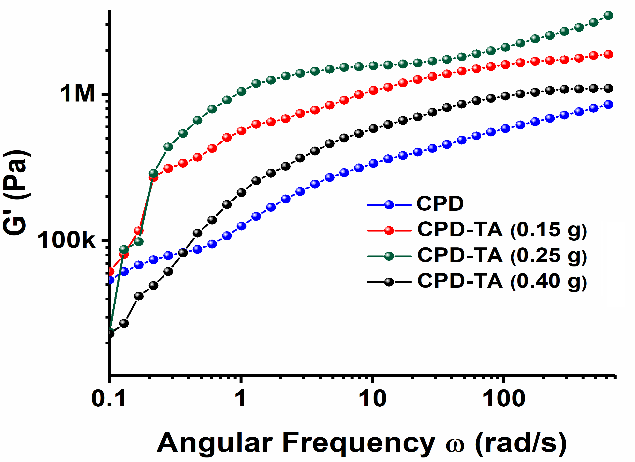
**

**Figure S24.** Rheological frequency sweeps where CPD-TA (0.25 g) shows maximum storage modulus.

**
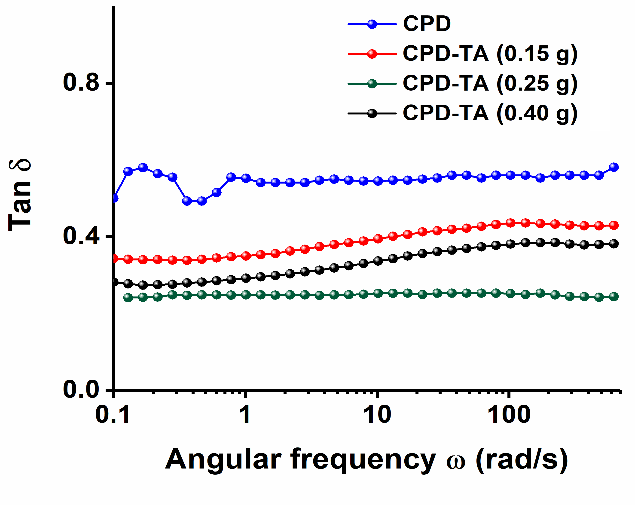
**

**Figure S25.** Tan δ values for different ratios of CPD-TA w.r.t angular frequency with the lowest value in CPD-TA (0.25 g).


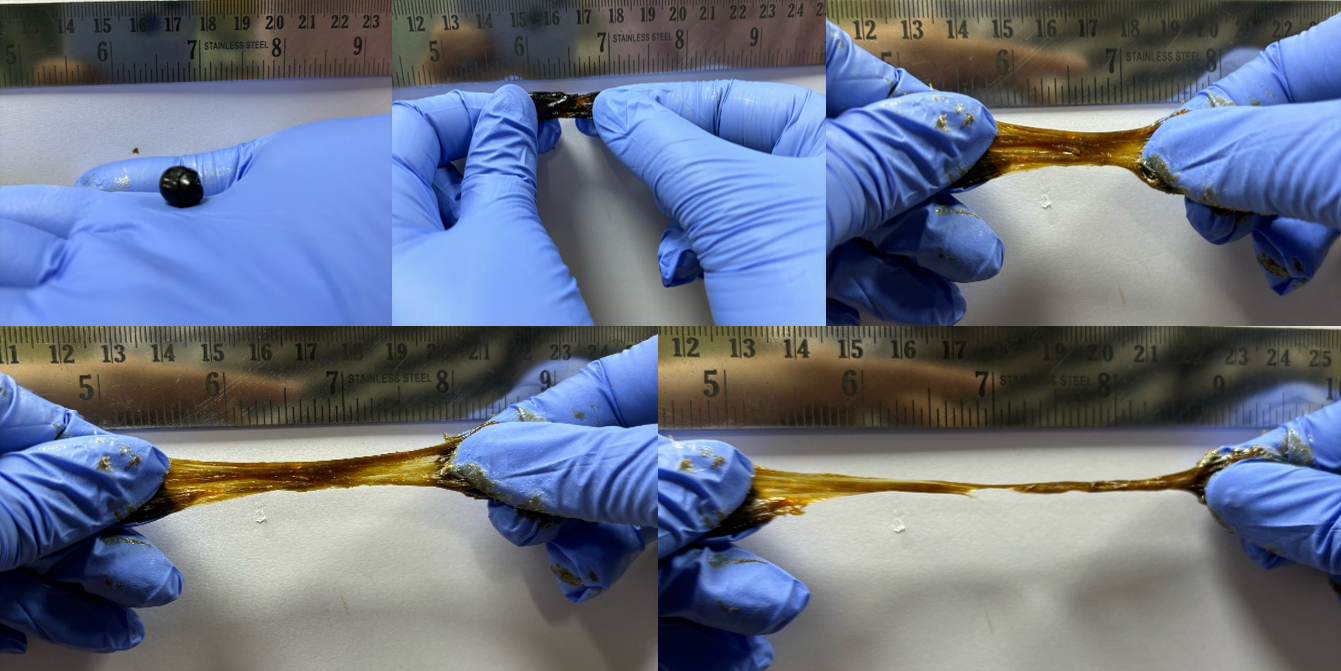


**Figure S26.** Self healed CPD-TA nanoglue stretchability upto an optimum extent.


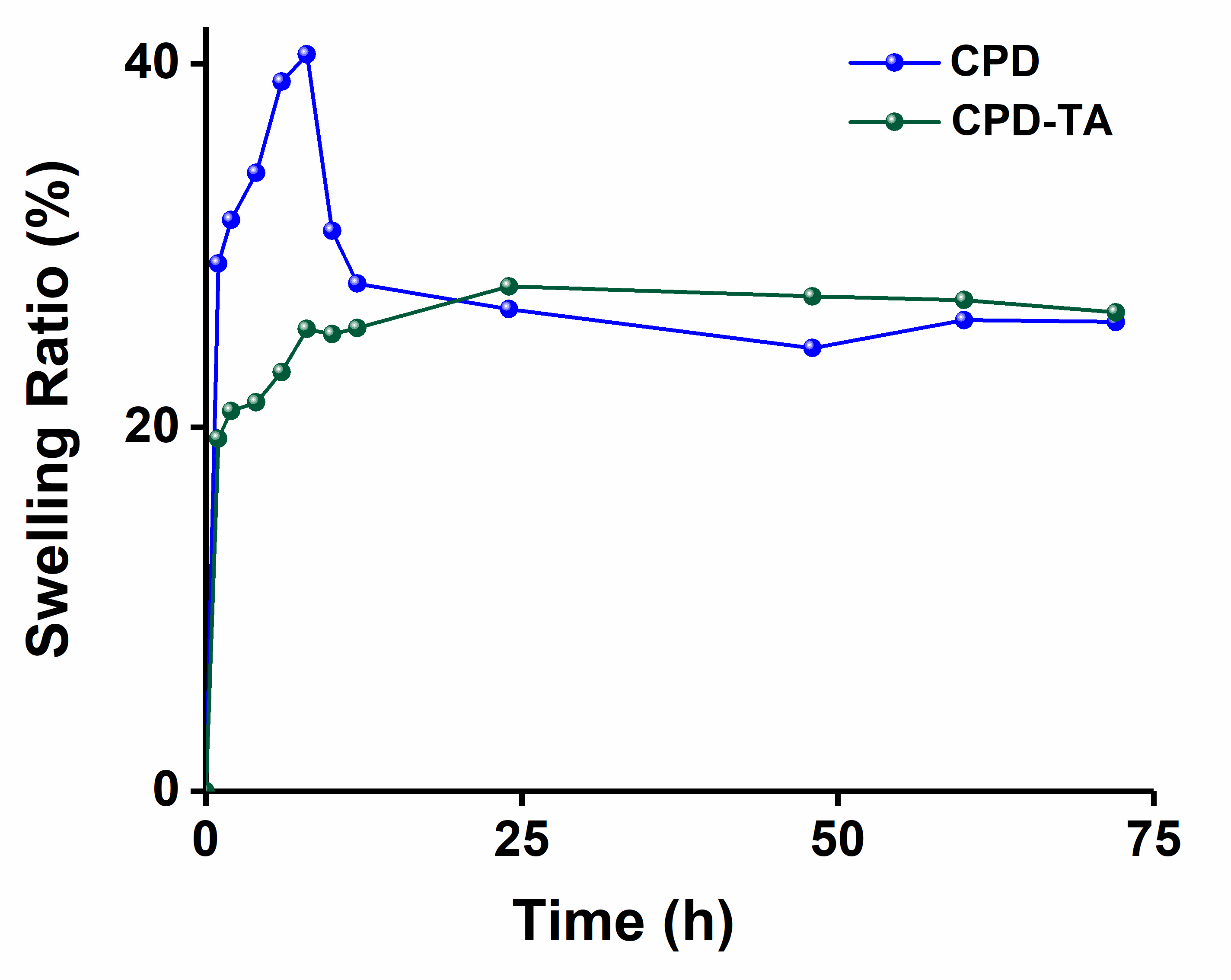


**Figure S27.** Swelling ratio of CPD (40%) and CPD-TA (27.74 %).

**
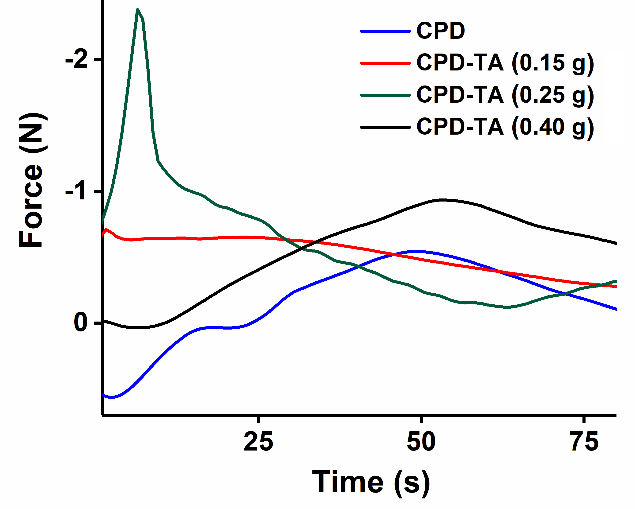
**

**Figure S28.** Probe tack test on different ratios of CPD-TA.

**
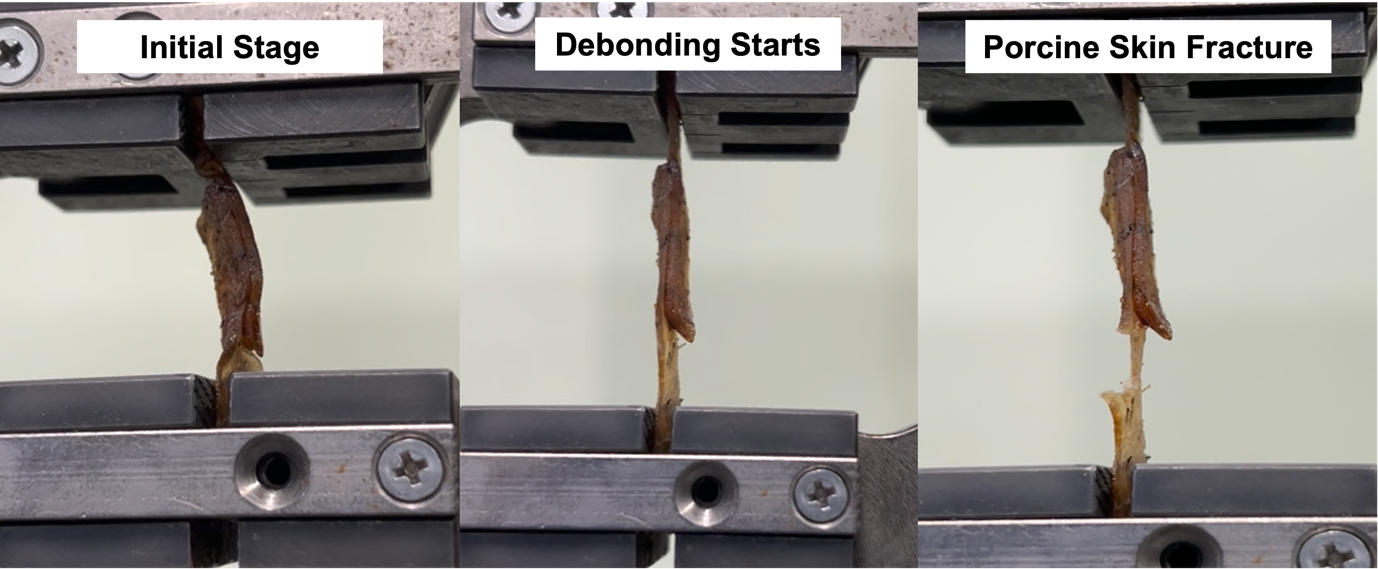
**

**Figure S29.** Digital image of bonding lap sheer in CPD-TA nanoglue.

**Table S2.** Comparison table of recent strength reported from lap sheer test.

| **S. No.** | **Formulations** | **Lap Sheer strength (MPa)** | **Reference** |
| --- | --- | --- | --- |
| 1. | CPD-TA nanoglue | 1.32 | This work |
| 2. | CD-Gelatin nanoadhesive | 0.57 | [1] |
| 3. | Gelatin/Chitosan/Carboxymethyl Cellulose | 0.055 | [2] |
| 4. | Pluronic F127-Lipoic Acid/ Ce^3+^/Tannic Acid/Ulinastatin nanohydrogel | 0.030 | [3] |
| 5. | PEG/TA/Gelatin | 0.020 | [4] |
| 6. | PEG/Gelatin Microgel | 0.004 | [5] |
| 7. | Gelatin Methacryloyl/Dopamine | 0.001 | [6] |
| 8. | Tannin/Gelatin hydrogel | 0.036 | [7] |
| 9. | PEGDA/TA | 0.128 | [8] |
| 10. | Polycation/TA | 0.300 | [9] |
| 11. | DNA/TA | 0.0035 | [10] |
| 12. | PAM/Glycerol/Gelatin/TA/Fe^3+^ | 0.098 | [11] |
| 13. | Gelatin/Hyaluronic Acid/TA | 0.048 | [12] |
| 14. | Collagen/TA | 0.045 | [13] |

**
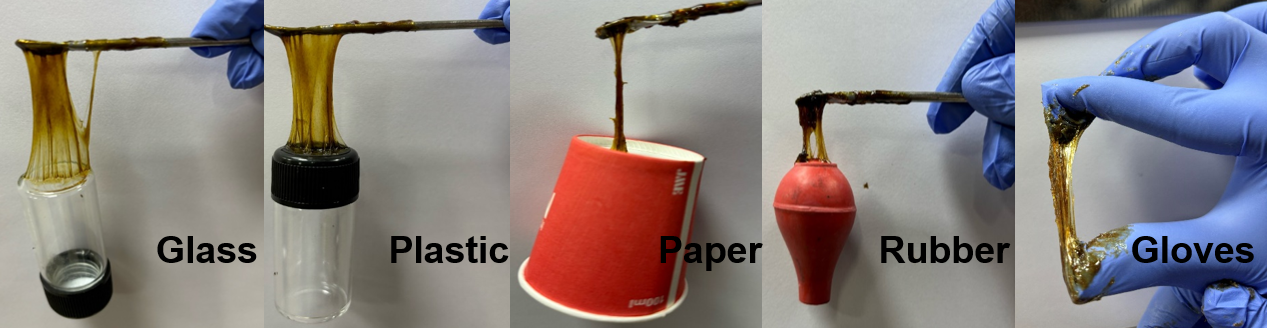
**

**Figure S30.** Nanoglue adheres to organic and inorganic substances like glass, plastic, paper, rubber and gloves.


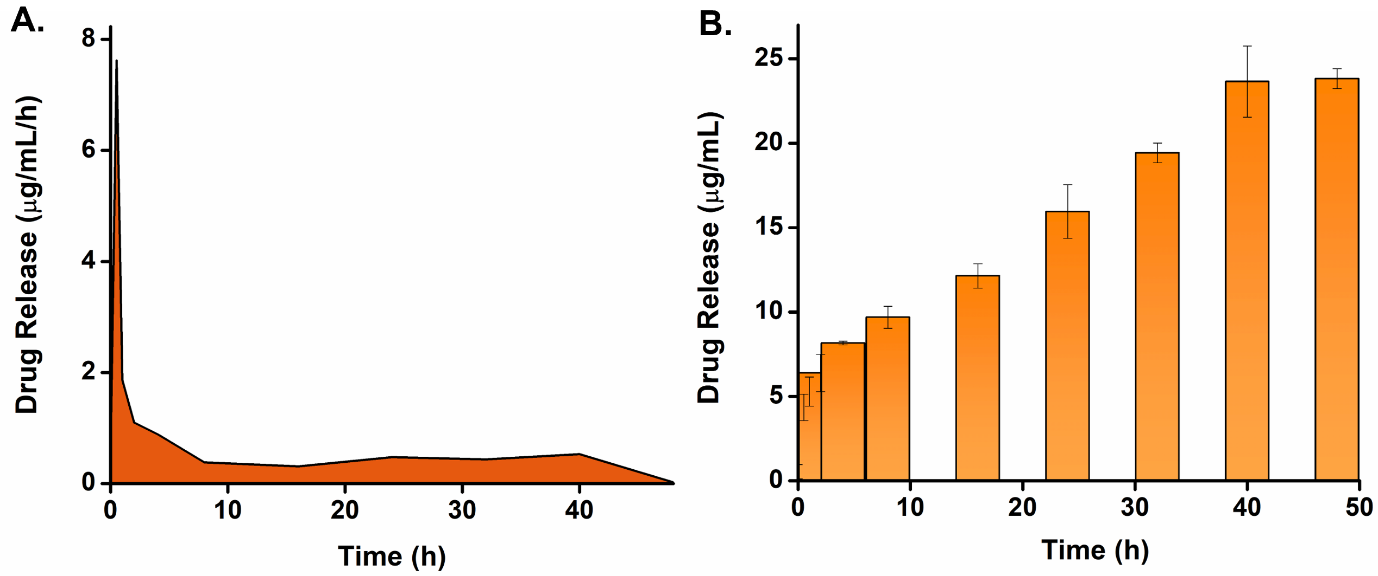


**Figure S31.** A. Plot shows the drug released per hour in mg/mL from CPD-TA:Ins and B. Plot shows the total drug released from the CPD-TA:Ins in mg/mL in 48 h.

**Table S3.** Comparative table of ROS measurement both in presence and absence of light for synthesized formulations.

| **In absence of light** | | | |
| --- | --- | --- | --- |
|  | **% total** | **Median value of FITC-A for DCFDA + representative population** | **Median value of FITC-A for whole population** |
| **Control** | 0.10 | 18069.51 | 17948.03 |
| **H_2_O_2_** | 16.10 | 14454.10 | 12468.48 |
| **CD** | 11.22 | 14382.66 | 13159.86 |
| **CPD** | 11.61 | 14055.76 | 11973.90 |
| **CPD-TA** | 9.76 | 14076.95 | 12301.39 |
| **Insulin** | 8.89 | 14273.22 | 12468.48 |
| **CPD-Ta:Ins** | 1.98 | 19330.51 | 12136.54 |
| **In presence of light** | | | |
| **CD** | 19.98 | 15399.17 | 12637.84 |
| **CPD** | 20.42 | 15103.74 | 12468.48 |
| **CPD-TA** | 27.90 | 16070.82 | 13338.61 |
| **Insulin** | 8.80 | 13983.58 | 12468.48 |
| **CPD-Ta:Ins** | 38.06 | 18908.78 | 18438.92 |

**
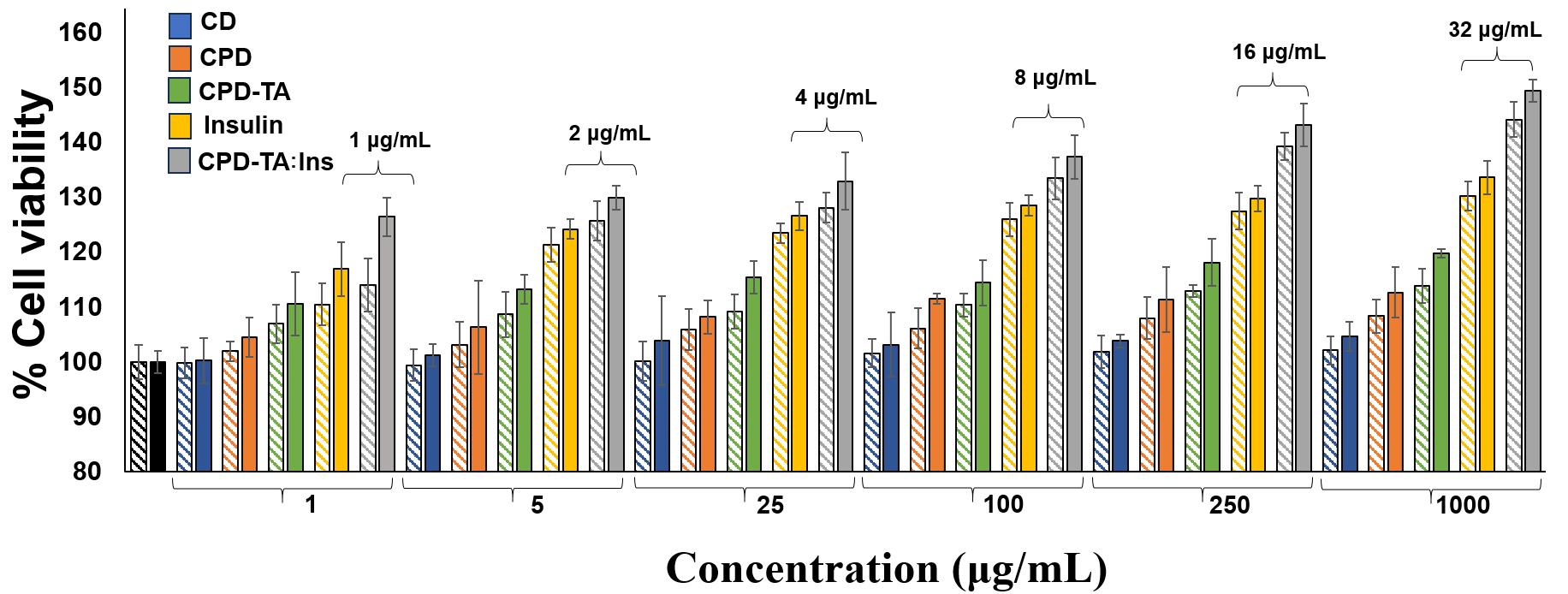
**

**Figure S32.** % Cell viability tested on HEKa cells without light with maximum for CPD-TA-Ins promote cell division and growth in normal wounds (solid bars) and diabetic wounds (patterned bars) for 24 h.

**
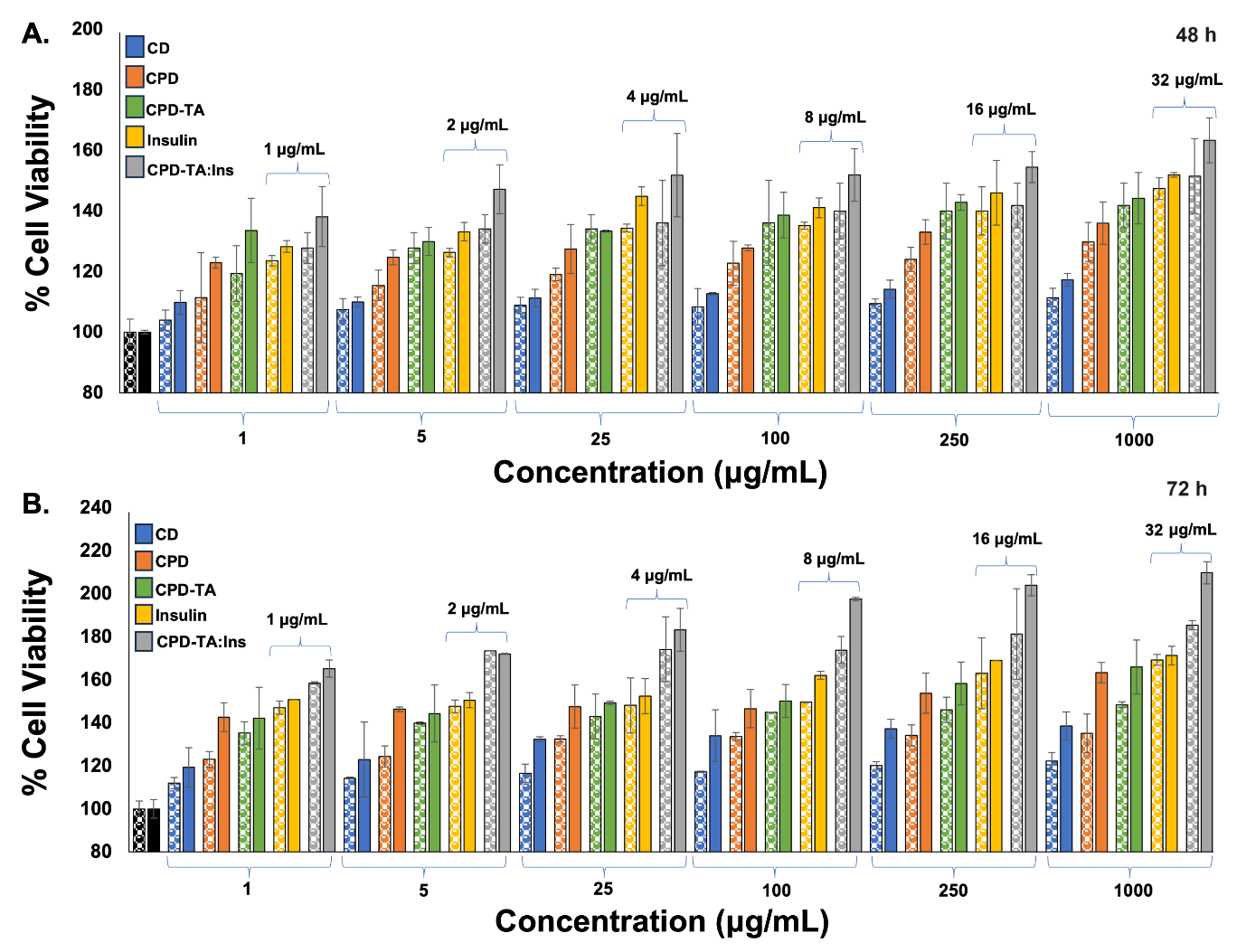
**

**Figure S33.** A-B. % Cell viability tested on HEKa cells without light with maximum for CPD-TA-Ins promote cell division and growth in normal wounds (solid bars) and diabetic wounds (patterned bars) for 48 and 72 h respectively.

**Table S4**. The table shows the % variation in mitochondrial reductase activity in the MTT assay for determining cellular metabolism rate using HEKa cells (normal cells) without light. The cells were treated with varying concentrations of CD, CPD, CPD-TA, and CPD-TA:Ins i.e., 1, 5, 25, 100, 250, and 1000 µg/mL respectively, for 24, 48 and 72 h. The insulin concentration used alone and in CPD-TA:Ins is 1, 2, 4, 8, 16, and 32 µg/mL respectively. The data were plotted as mean value ± SD of three independent experiments.

| **24 h** | | | | | | |
| --- | --- | --- | --- | --- | --- | --- |
| **Dose (µg/mL)** | **CD (%)** | **CPD (%)** | **CPD-TA (%)** | **Dose**  **(µg/mL)** | **Insulin (%)** | **CPD-TA:Ins (%)** |
| **1** | 100.27 ± 4.13 | 104.50 ± 3.59 | 110.57 ± 5.78 | **1** | 116.93 ± 4.88 | 126.41 ± 3.56 |
| **5** | 101.30 ± 2.02 | 106.32 ± 8.56 | 113.27 ± 2.67 | **2** | 124.20 ± 1.79 | 129.89 ± 2.16 |
| **25** | 103.87 ± 8.11 | 108.21 ± 3.01 | 115.45 ± 2.94 | **4** | 126.57 ± 2.53 | 132.89 ± 5.22 |
| **100** | 103.19 ± 5.90 | 111.48 ± 0.92 | 114.42 ± 4.11 | **8** | 128.51 ± 1.92 | 137.36 ± 3.95 |
| **250** | 103.86 ± 1.17 | 111.40 ± 5.87 | 118.11 ± 4.28 | **16** | 129.83 ± 2.33 | 143.10 ± 3.89 |
| **1000** | 104.69 ± 2.71 | 112.67 ± 4.63 | 119.79 ± 0.84 | **32** | 133.59 ± 3.09 | 149.39 ± 1.98 |
| **48 h** | | | | | | |
| **1** | 109.79 ± 3.88 | 122.94 ± 1.83 | 133.58 ± 10.61 | **1** | 128.26 ± 1.90 | 138.12 ± 9.93 |
| **5** | 109.98 ± 1.62 | 124.68 ± 2.41 | 129.91 ± 4.57 | **2** | 133.14 ± 3.07 | 147.20 ± 8.07 |
| **25** | 111.28 ± 2.89 | 127.42 ± 8.06 | 133.30 ± 0.35 | **4** | 144.86 ± 3.04 | 151.77 ± 13.74 |
| **100** | 112.68 ± 0.42 % | 127.79 ± 0.95 | 138.61 ± 7.49 | **8** | 141.04 ± 3.25 | 151.86 ± 8.65 |
| **250** | 114.17 ± 2.96 | 133.02 ± 4.04 | 142.81 ± 2.61 | **16** | 145.98 ± 10.67 | 154.47 ± 5.09 |
| **1000** | 117.22 ± 2.10 | 135.94 ± 6.99 | 144.21 ± 8.51 | **32** | 151.92 ± 0.75 | 163.33 ± 7.42 |
| **72 h** | | | | | | |
| **1** | 119.29 ± 9.19 | 142.64 ± 6.64 | 142.12 ± 14.39 | **1** | 150.81 ± 0.07 | 165.22 ± 2.43 |
| **5** | 122.91 ± 17.46 | 146.37 ± 1.06 | 144.29 ± 13.29 | **2** | 150.53 ± 3.46 | 172.03 ± 0.28 |
| **25** | 132.40 ± 1.13 | 147.60 ± 9.99 | 149.30 ± 0.77 | **4** | 152.44 ± 8.20 | 183.25 ± 9.89 |
| **100** | 134.05 ± 11.99 | 146.60 ± 8.90 | 150.20 ± 7.65 | **8** | 162.10 ± 1.83 | 197.58 ± 0.84 |
| **250** | 137.19 ± 4.55 | 153.78 ± 9.40 | 158.25 ± 9.97 | **16** | 168.98 ± 0.07 | 203.96 ± 4.85 |
| **1000** | 138.58 ± 6.46 | 163.34 ± 4.73 | 165.89 ± 12.56 | **32** | 171.26 ± 4.26 | 209.75 ± 5.19 |

**Table S5**. The table shows the % variation in mitochondrial reductase activity in the MTT assay for determining cellular metabolism rate using HEKa cells (diabetic cells) without light. The cells were treated with varying concentrations of CD, CPD, CPD-TA, and CPD-TA:Ins, i.e., 1, 5, 25, 100, 250, and 1000 µg/mL respectively, for 24, 48, 72 h. The insulin concentration used alone and in CPD-TA:Ins is 1, 2, 4, 8, 16, and 32 µg/mL respectively. The data were plotted as mean value ± SD of three independent experiments.

| **24 h (Diabetic Conditions)** | | | | | | |
| --- | --- | --- | --- | --- | --- | --- |
| **Dose**  **(µg/mL)** | **CD (%)** | **CPD (%)** | **CPD-TA (%)** | **Dose**  **(µg/mL)** | **Insulin (%)** | **CPD-TA:Ins (%)** |
| **1** | 99.88 ± 2.82 | 101.97 ± 1.78 | 106.94 ± 3.55 | **1** | 110.49 ± 3.86 | 114.02 ± 4.88 |
| **5** | 99.39 ± 2.88 | 103.15 ± 4.14 | 108.67 ± 4.13 | **2** | 121.39 ± 3.12 | 125.73 ± 3.56 |
| **25** | 100.16 ± 3.53 | 105.91 ± 3.71 | 109.19 ± 3.13 | **4** | 123.47 ± 1.82 | 128.10 ± 2.69 |
| **100** | 101.60 ± 2.59 | 106.13 ± 3.70 | 110.40 ± 2.07 | **8** | 125.94 ± 3.04 | 133.44 ± 3.79 |
| **250** | 101.88 ± 2.88 | 108.01 ± 3.87 | 112.93 ± 1.04 | **16** | 127.48 ± 3.37 | 139.29 ± 2.49 |
| **1000** | 102.14 ± 2.55 | 108.40 ± 3.02 | 113.92 ± 3.11 | **32** | 130.25 ± 2.65 | 144.16 ± 3.21 |
| **48 h** | | | | | | |
| **1** | 103.94 ± 3.32 | 111.36 ± 14.84 | 119.38 ± 9.18 | **1** | 123.57 ± 1.70 | 127.76 ± 5.09 |
| **5** | 107.44 ± 3.60 | 115.43 ± 5.03 | 127.76 ± 5.09 | **2** | 126.30 ± 1.35 | 134.05 ± 4.73 |
| **25** | 108.80 ± 2.68 | 119.01 ± 2.12 | 134.05 ± 4.73 | **4** | 134.32 ± 1.27 | 136.05± 14.07 |
| **100** | 108.35 ± 6.01 | 122.75 ± 7.28 | 136.05±14.07 | **8** | 135.14 ± 1.20 | 139.97 ± 9.19 |
| **250** | 109.35 ± 1.55 | 123.99 ± 3.95 | 139.97 ± 9.19 | **16** | 139.97 ± 7.40 | 141.79 ± 7.40 |
| **1000** | 111.36 ± 3.11 | 129.76 ± 6.39 | 141.79 ± 7.42 | **32** | 147.35 ± 3.60 | 151.51± 12.36 |
| **72 h** | | | | | | |
| **1** | 111.99 ± 2.68 | 123.06 ± 3.67 | 135.27 ± 5.23 | **1** | 147.06 ± 3.11 | 158.48 ± 0.56 |
| **5** | 114.33 ± 0.35 | 124.41 ± 4.87 | 139.88 ± 0.49 | **2** | 147.73 ± 2.90 | 173.53 ± 0.07 |
| **25** | 116.53 ± 4.24 | 132.50 ± 1.48 | 143.00±10.39 | **4** | 148.14 ± 12.83 | 174.15± 15.06 |
| **100** | 117.17 ± 0.35 | 133.64 ± 1.90 | 144.85 ± 0.07 | **8** | 149.58 ± 0.20 | 173.84 ± 6.29 |
| **250** | 120.15 ± 1.76 | 134.20 ± 4.87 | 146.13 ± 5.86 | **16** | 163.04 ± 16.47 | 181.32± 21.00 |
| **1000** | 122.21 ± 3.99 | 135.13 ± 9.05 | 148.40 ± 1.34 | **32** | 169.29 ± 2.54 | 185.39 ± 2.02 |

**Table S6**. The table shows the % variation in mitochondrial reductase activity in the MTT assay for determining cellular metabolism rate using HEKa cells (normal cells) with light. The cells were treated with varying concentrations of CD, CPD, CPD-TA, and CPD-TA:Ins, i.e., 1, 5, 25, 100, 250, and 1000 µg/mL respectively, for 24 h. The insulin concentration used alone and in CPD-TA:Ins is 1, 2, 4, 8, 16, and 32 µg/mL respectively. The data were plotted as mean value ± SD of three independent experiments.

| **Dose**  **(µg/mL)** | **CD (%)** | **CPD (%)** | **CPD-TA (%)** | **Dose (µg/mL)** | **Insulin (%)** | **CPD-TA:Ins (%)** |
| --- | --- | --- | --- | --- | --- | --- |
| **1** | 98.22 ± 2.41 | 103.55 ± 4.15 | 110.31 ± 3.74 | **1** | 117.90 ± 4.88 | 126.61 ± 4.49 |
| **5** | 100.99 ± 4.22 | 105.47 ± 3.12 | 114.00 ± 5.34 | **2** | 122.17 ± 4.76 | 130.94 ± 2.66 |
| **25** | 102.52 ± 3.29 | 107.32 ± 4.26 | 115.89 ± 2.94 | **4** | 125.56 ± 2.43 | 132.03 ± 2.58 |
| **100** | 103.12 ± 4.31 | 109.84 ± 4.59 | 118.06 ± 1.62 | **8** | 126.36 ± 2.93 | 136.30 ± 5.61 |
| **250** | 103.32 ± 2.78 | 111.63 ± 4.00 | 118.83 ± 1.61 | **16** | 125.49 ± 4.83 | 144.58 ± 2.98 |
| **1000** | 104.84 ± 3.08 | 112.29 ± 5.50 | 120.48 ± 4.93 | **32** | 129.40 ± 6.31 | 148.74 ± 4.14 |

**Table S7**. The table shows the % variation in mitochondrial reductase activity in the MTT assay for determining cellular metabolism rate using HEKa cells (diabetic cells) with light. The cells were treated with varying concentrations of CD, CPD, CPD-TA, and CPD-TA:Ins, i.e., 1, 5, 25, 100, 250, and 1000 µg/mL respectively, for 24 h. The insulin concentration used alone and in CPD-TA:Ins is 1, 2, 4, 8, 16, and 32 µg/mL respectively. The data were plotted as mean value ± SD of three independent experiments.

| **Dose**  **(µg/mL)** | **CD (%)** | **CPD (%)** | **CPD-TA (%)** | **Dose (µg/mL)** | **Insulin (%)** | **CPD-TA:Ins (%)** |
| --- | --- | --- | --- | --- | --- | --- |
| **1** | 100.47 ± 3.02 | 100.61 ± 2.90 | 105.93 ± 4.22 | **1** | 111.08 ± 2.98 | 114.78 ± 3.48 |
| **5** | 100.76 ± 3.88 | 101.61 ± 2.67 | 108.71 ± 2.66 | **2** | 118.22 ± 1.81 | 121.13 ± 3.50 |
| **25** | 101.39 ± 3.64 | 103.93 ± 3.46 | 109.69 ± 3.02 | **4** | 122.03 ± 4.87 | 123.88 ± 3.60 |
| **100** | 102.25 ± 3.37 | 104.69 ± 3.70 | 110.47 ± 3.41 | **8** | 123.68 ± 3.04 | 131.46 ± 3.92 |
| **250** | 102.57 ± 3.97 | 106.32 ± 1.84 | 111.78 ± 2.41 | **16** | 125.49 ± 3.29 | 137.93 ± 2.91 |
| **1000** | 102.45 ± 1.48 | 107.63 ± 1.43 | 113.43 ± 3.11 | **32** | 129.40 ± 3.26 | 142.64 ± 3.85 |

**Table S8.** It shows the p values calculated for % variation in cell viability in normal and diabetic conditions (without light) after treatment with varying concentration of CD, CPD, CPD-TA, and CPD-TA:Ins, that is, i.e., 1, 5, 25, 100, 250, and 1000 µg/mL respectively, for 24 h. The insulin concentration used alone and in CPD-TA:Ins is 1, 2, 4, 8, 16, and 32 µg/mL respectively. The statistical significance of data is considered when p < 0.05.

| **p-value for checking the statistical significance of cell viability in normal conditions (24h)** | | | | | | |
| --- | --- | --- | --- | --- | --- | --- |
| **Dose**  **(µg/mL)** | **CD** | **CPD** | **CPD-TA** | **Dose (µg/mL)** | **Insulin** | **CPD-TA:Ins** |
| **1** | 0.874019 | 0.536566 | 0.135347 | **1** | 0.001024 | 0.016531 |
| **5** | 0.931491 | 0.590236 | 0.022504 | **2** | 0.013501 | 0.007108 |
| **25** | 0.728072 | 0.263828 | 0.016892 | **4** | 0.011842 | 0.01384 |
| **100** | 0.723129 | 0.104304 | 0.045214 | **8** | 0.007895 | 0.005681 |
| **250** | 0.523097 | 0.245887 | 0.025114 | **16** | 0.007895 | 0.003296 |
| **1000** | 0.488438 | 0.162012 | 0.022358 | **32** | 0.006185 | 0.00105 |
| **p-value for checking the statistical significance of cell viability in diabetic conditions (24h)** | | | | | | |
| **Dose**  **(µg/mL)** | **CD** | **CPD** | **CPD-TA** | **Dose (µg/mL)** | **Insulin** | **CPD-TA:Ins** |
| **1** | 0.978286 | 0.533803 | 0.19874 | **1** | 0.009141 | 0.002848 |
| **5** | 0.748748 | 0.559771 | 0.15443 | **2** | 0.007091 | 0.004732 |
| **25** | 0.656332 | 0.271181 | 0.095242 | **4** | 0.002423 | 0.002031 |
| **100** | 0.537403 | 0.255606 | 0.043467 | **8** | 0.00337 | 0.002049 |
| **250** | 0.516944 | 0.167074 | 0.029728 | **16** | 0.003326 | 0.000497 |
| **1000** | 0.459477 | 0.112609 | 0.029728 | **32** | 0.001467 | 0.000496 |

**Table S9.** It shows the p values calculated for % variation in cell viability in normal and diabetic conditions (without light) after treatment with varying concentration of CD, CPD, CPD-TA, and CPD-TA:Ins, that is, i.e., 1, 5, 25, 100, 250, and 1000 µg/mL respectively, for 48 h. The insulin concentration used alone and in CPD-TA:Ins is 1, 2, 4, 8, 16, and 32 µg/mL respectively. The statistical significance of data is considered when p < 0.05.

| **p-value for checking the statistical significance of cell viability in normal conditions (48h)** | | | | | | |
| --- | --- | --- | --- | --- | --- | --- |
| **Dose**  **(µg/mL)** | **CD** | **CPD** | **CPD-TA** | **Dose (µg/mL)** | **Insulin** | **CPD-TA:Ins** |
| **1** | 0.070253 | 0.00065 | 0.079842 | **1** | 0.002231 | 0.023731 |
| **5** | 0.054899 | 0.000801 | 0.003873 | **2** | 0.000618 | 0.005662 |
| **25** | 0.059095 | 0.080861 | 0.02849 | **4** | 0.000362 | 0.045883 |
| **100** | 0.040164 | 2.61E-05 | 0.003836 | **8** | 0.021127 | 0.010178 |
| **250** | 0.01924 | 0.001718 | 0.033946 | **16** | 0.005509 | 0.000554 |
| **1000** | 0.001957 | 0.009005 | 0.008603 | **32** | 1.25E-06 | 0.007575 |
| **p-value for checking the statistical significance of cell viability in diabetic conditions (48h)** | | | | | | |
| **Dose**  **(µg/mL)** | **CD** | **CPD** | **CPD-TA** | **Dose (µg/mL)** | **Insulin** | **CPD-TA:Ins** |
| **1** | 0.53177 | 0.241511 | 0.144245 | **1** | 0.008783 | 0.021297 |
| **5** | 0.166372 | 0.092612 | 0.093254 | **2** | 0.005432 | 0.029642 |
| **25** | 0.115564 | 0.031383 | 0.012398 | **4** | 0.011603 | 0.072113 |
| **100** | 0.176707 | 0.027825 | 0.055558 | **8** | 0.024824 | 0.020504 |
| **250** | 0.097581 | 0.017969 | 0.020094 | **16** | 0.013605 | 0.009863 |
| **1000** | 0.098572 | 0.021684 | 0.019038 | **32** | 0.005838 | 0.020238 |

**Table S10.** It shows the p values calculated for % variation in cell viability in normal and diabetic conditions (without light) after treatment with varying concentration of CD, CPD, CPD-TA, and CPD-TA:Ins, that is, i.e., 1, 5, 25, 100, 250, and 1000 µg/mL respectively, for 72q h. The insulin concentration used alone and in CPD-TA:Ins is 1, 2, 4, 8, 16, and 32 µg/mL respectively. The statistical significance of data is considered when p < 0.05.

| **p-value for checking the statistical significance of cell viability in normal conditions (72h)** | | | | | | |
| --- | --- | --- | --- | --- | --- | --- |
| **Dose**  **(µg/mL)** | **CD** | **CPD** | **CPD-TA** | **Dose (µg/mL)** | **Insulin** | **CPD-TA:Ins** |
| **1** | 0.070253 | 0.003744 | 0.089379 | **1** | 0.005037 | 0.006724 |
| **5** | 0.054899 | 0.079448 | 0.03369 | **2** | 0.009723 | 0.002531 |
| **25** | 0.059095 | 0.012466 | 0.0022 | **4** | 0.028166 | 0.015755 |
| **100** | 0.040164 | 0.013028 | 0.003033 | **8** | 0.004253 | 0.001449 |
| **250** | 0.01924 | 0.009702 | 0.014235 | **16** | 0.002743 | 0.004471 |
| **1000** | 0.001957 | 0.002065 | 0.005088 | **32** | 0.002902 | 0.003469 |
| **p-value for checking the statistical significance of cell viability in diabetic conditions (72h)** | | | | | | |
| **Dose**  **(µg/mL)** | **CD** | **CPD** | **CPD-TA** | **Dose (µg/mL)** | **Insulin** | **CPD-TA:Ins** |
| **1** | 0.120499 | 0.046732 | 0.031178 | **1** | 0.000574 | 0.001317 |
| **5** | 0.225013 | 0.05695 | 0.008286 | **2** | 0.000496 | 0.00066 |
| **25** | 0.001401 | 0.014194 | 0.060048 | **4** | 0.007847 | 0.006703 |
| **100** | 0.039942 | 0.0145 | 0.006451 | **8** | 0.000206 | 0.008626 |
| **250** | 0.00263 | 0.030258 | 0.021732 | **16** | 0.021587 | 9.98E-05 |
| **1000** | 0.005063 | 0.069501 | 0.006308 | **32** | 0.001 | 3.57E-05 |

**Table S11.** It shows the p values calculated for % variation in cell viability in normal and diabetic conditions (with light) after treatment with varying concentrations of CD, CPD, CPD-TA, and CPD-TA:Ins, that is, i.e., 1, 5, 25, 100, 250, and 1000 µg/mL respectively, for 24 h. The insulin concentration used alone and in CPD-TA:Ins is 1, 2, 4, 8, 16, and 32 µg/mL respectively. The statistical significance of data is considered when p < 0.05.

| **p-value for checking the statistical significance of cell viability in normal conditions** | | | | | | |
| --- | --- | --- | --- | --- | --- | --- |
| **Dose**  **(µg/mL)** | **CD** | **CPD** | **CPD-TA** | **Dose (µg/mL)** | **Insulin** | **CPD-TA:Ins** |
| **1** | 0.679057 | 0.567661 | 0.125408 | **1** | 0.056468 | 0.011891 |
| **5** | 0.627665 | 0.304398 | 0.116744 | **2** | 0.025485 | 0.001822 |
| **25** | 0.627665 | 0.277773 | 0.006747 | **4** | 0.003088 | 0.001495 |
| **100** | 0.624084 | 0.187202 | 0.00567 | **8** | 0.004112 | 0.007742 |
| **250** | 0.485491 | 0.104135 | 0.004839 | **16** | 0.012788 | 0.000591 |
| **1000** | 0.352262 | 0.163184 | 0.035479 | **32** | 0.016574 | 0.001028 |
| **p-value for checking the statistical significance of cell viability in diabetic conditions** | | | | | | |
| **Dose**  **(µg/mL)** | **CD** | **CPD** | **CPD-TA** | **Dose (µg/mL)** | **Insulin** | **CPD-TA:Ins** |
| **1** | 0.909988 | 0.88036 | 0.276565 | **1** | 0.046586 | 0.024714 |
| **5** | 0.872447 | 0.686098 | 0.078426 | **2** | 0.005113 | 0.007461 |
| **25** | 0.762275 | 0.401875 | 0.068801 | **4** | 0.013247 | 0.005095 |
| **100** | 0.615355 | 0.299295 | 0.065867 | **8** | 0.008543 | 0.00222 |
| **250** | 0.601085 | 0.126936 | 0.02988 | **16** | 0.003369 | 0.000596 |
| **1000** | 0.47832 | 0.07078 | 0.037295 | **32** | 0.001951 | 0.000669 |

**Table S12.** It shows the comparative data of variation in wound diameter in diabetic and normal conditions after treatment with 1000 µg/mL of CD, CPD, CPD-TA, insulin, and CPD-TA:Ins and the concentration of insulin used is 32 µg/mL. The data was measured after a time duration of 6, 12, and 24 h.

| **% change in normal wound diameter with time** | | | | | |
| --- | --- | --- | --- | --- | --- |
| **Time (h)** | **CD (%)** | **CPD (%)** | **CPD-TA (%)** | **Insulin (%)** | **CPD-TA:Ins (%)** |
| **6** | 5.39 ± 1.01 | 11.90 ± 0.72 | 23.17 ± 0.57 | 28.25 ± 1.12 | 39.52 ± 0.54 |
| **12** | 12.30 ± 1.32 | 24.61 ± 1.18 | 29.23 ± 0.80 | 40.00 ± 0.64 | 47.69 ± 0.50 |
| **24** | 21.21 ± 0.45 | 26.52 ± 0.85 | 33.42 ± 0.80 | 41.11 ± 0.25 | 59.41 ± 0.75 |
| **% change in diabetic wound diameter with time** | | | | | |
| **Time (h)** | **CD (%)** | **CPD (%)** | **CPD-TA (%)** | **Insulin (%)** | **CPD-TA:Ins (%)** |
| **6** | 2.611 ± 0.66 | 10.44 ± 0.66 | 19.77 ± 1.37 | 26.49 ± 1.37 | 38.806 ± 0.62 |
| **12** | 9.17 ± 1.25 | 19.26 ± 0.57 | 25.22 ± 0.72 | 34.40 ± 0.68 | 44.72 ± 0.68 |
| **24** | 15.77 ± 0.57 | 23.52 ± 0.68 | 29.14 ± 0.36 | 38.23 ± 0.33 | 50.53 ± 0.66 |

**Table S13.** It shows the p values calculated for % variation in wound diameter in normal and diabetic conditions after treatment with 1000 µg/mL of CD, CPD, CPD-TA, insulin, and CPD-TA:Ins and the concentration of insulin used is 32 µg/mL. The statistical significance of data is considered when p < 0.05.

| **p-value for checking the statistical significance of wound healing in normal conditions** | | | | | |
| --- | --- | --- | --- | --- | --- |
| **Time (h)** | **CD** | **CPD** | **CPD-TA** | **Insulin** | **CPD-TA:Ins** |
| **6** | 0.201555 | 0.017948 | 0.001567 | 0.001583 | 0.000192 |
| **12** | 0.037474 | 0.002622 | 0.000529 | 0.000102 | 3.47E-05 |
| **24** | 0.001811 | 0.00248 | 0.001146 | 0.000146 | 8.13E-05 |
| **p-value for checking the statistical significance of wound healing in diabetic conditions** | | | | | |
| **Time (h)** | **CD** | **CPD** | **CPD-TA** | **Insulin** | **CPD-TA:Ins** |
| **6** | 0.360342 | 0.014513 | 0.007924 | 0.002743 | 9.67E-05 |
| **12** | 0.116117 | 0.002794 | 0.001473 | 0.000409 | 0.000146 |
| **24** | 0.012161 | 0.00378 | 0.000836 | 0.000276 | 7.96E-05 |

**Table S14.** The Combination Index (CI) values for the cell viability for varying combinations of CPD-TA with insulin protein were calculated to check if the two drugs are synergistic or antagonistic, and the values come out to be less than 1, indicating the synergistic effect of drugs.

| **Combination Index (CI)** | | | | |
| --- | --- | --- | --- | --- |
| **Concentration of CPD-TA used (µg/mL)** | **Concentration of insulin used (µg/mL)** | **D_x1_**  **(CPD-TA) = D_m_ [f_a_/f_u_]^1/m^** | **D_x2_ (Insulin) = D_m_ [f_a_/f_u_]^1/m^** | **CI = (D_1_) /(D_x1_) + (D_2_)/( D_x2_)** |
| 1 | 1 | 14547574 | 2212.948 | 0.00045195 |
| 5 | 2 | 1105467 | 273.1131 | 0.0073275 |
| 25 | 4 | 623103.4 | 229.6153 | 0.01746056 |
| 100 | 8 | 207358.8 | 193.8836 | 0.04174413 |
| 250 | 16 | 41574.3 | 177.2352 | 0.09628883 |
| 1000 | 32 | 26150.43 | 154.3763 | 0.24552605 |


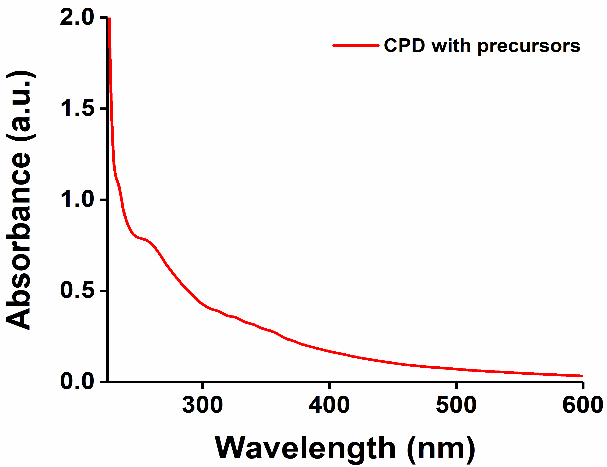


**Figure S34.** UV-Visible spectra of CPD with organic precursors.


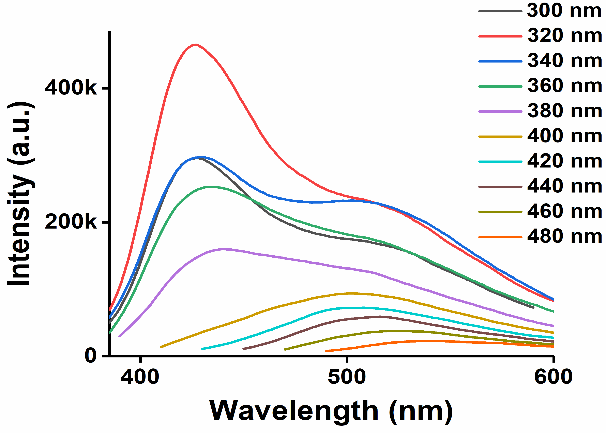


**Figure S35.** Steady-state fluorescence spectra of CPD with organic precursors.

**
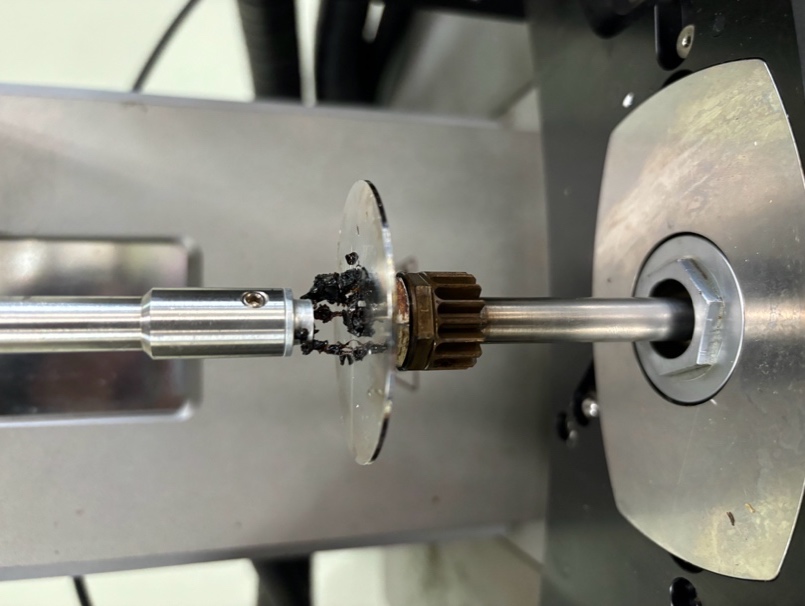
**

**Figure S36.** Low adhesion quality of CPD with organic precursors.

**References**

[1] M. Aggarwal, H. Panigrahi, D. K. Kotnees, P. Das, *Biomacromolecules* **2024**, *25*, 3178.

[2] M. Pan, T. Shui, Z. Zhao, M. Li, H. Fan, J. Wu, H. Zeng, *Chem. Mater.* **2023**, *35*, 4998.

[3] T. Wang, H. Fang, S. Yalikun, J. Li, Y. Pan, K. Zhang, J. Yin, H. Cui, *Biomacromolecules* **2024**, *25*, 924.

[4] H. Li, Y. Shi, W. Zhang, M. Yu, X. Chen, M. Kong, *ACS Appl. Mater. Interfaces* **2022**, *14*, 18097.

[5] Y. Li, H. Meng, Y. Liu, A. Narkar, B. P. Lee, *ACS Appl. Mater. Interfaces* **2016**, *8*, 11980.

[6] H. Montazerian, A. Baidya, R. Haghniaz, E. Davoodi, S. Ahadian, N. Annabi, A. Khademhosseini, P. S. Weiss, *ACS Appl. Mater. Interfaces* **2021**, *13*, 40290.

[7] Q. Zhao, S. Mu, Y. Long, J. Zhou, W. Chen, D. Astruc, C. Gaidau, H. Gu, *Macromol. Mater. Eng.* **2019**, *304*, 1.

[8] K. Chen, Q. Lin, L. Wang, Z. Zhuang, Y. Zhang, D. Huang, H. Wang, *ACS Appl. Mater. Interfaces* **2021**, *13*, 9748.

[9] Z. Wang, S. Zhang, S. Zhao, H. Kang, Z. Wang, C. Xia, Y. Yu, J. Li, *Chem. Eng. J.* **2021**, *404*, 127069.

[10] M. Shin, J. H. Ryu, J. P. Park, K. Kim, J. W. Yang, H. Lee, *Adv. Funct. Mater.* **2015**, *25*, 1270.

[11] X. Guo, W. Qin, C. Gu, X. Li, M. Chen, H. Zhai, X. Zhao, H. Liu, B. Zhao, Y. Zhang, Y. Wang, S. Yin, *Adv. Mater. Technol.* **2024**, *9*, 1.

[12] H. Wang, M. Wang, J. Wu, S. Zhu, Y. Ye, Y. Liu, K. Li, R. Li, Y. Zhang, M. Wei, X. Yang, L. Meng, *Adv. Healthc. Mater.* **2024**, *2304444*, 1.

[13] L. Shang, Y. Yan, Z. Li, H. Liu, S. Ge, B. Ma, *Adv. Sci.* **2024**, *11*, 1.
